# The neural correlates of response inhibition across the transition from infancy to toddlerhood: An fNIRS study

**DOI:** 10.1162/imag_a_00206

**Published:** 2024-06-28

**Authors:** Abigail Fiske, Liam Collins-Jones, Carina de Klerk, Katie Y.K. Lui, Alexandra Hendry, Isobel Greenhalgh, Anna Hall, Henrik Dvergsdal, Gaia Scerif, Karla Holmboe

**Affiliations:** Department of Experimental Psychology, University of Oxford, Oxford, United Kingdom; Department of Clinical Neurosciences, University of Cambridge, Cambridge, United Kingdom; Department of Psychology, University of Essex, Colchester, Essex, United Kingdom; Faculty of Education, University of Cambridge, Cambridge, United Kingdom; Department of Psychology, University of Cambridge, Cambridge, United Kingdom; Institute of Mental Health, University College London, London, United Kingdom; Nord University Business School, Department of Entrepreneurship, Innovation and Organisation, Bodø, Norway; School of Psychological Science, University of Bristol, Bristol, United Kingdom

**Keywords:** executive function, inhibitory control, response inhibition, infancy, toddlerhood, functional near-infrared spectroscopy, fNIRS, prefrontal cortex, PFC, parietal cortex

## Abstract

The transition from late infancy into toddlerhood represents a fundamental period in early development. During this time, the prefrontal cortex (PFC) is undergoing structural and functional maturation processes that parallel the emergence and improvement of executive function skills, such as inhibitory control. Despite the importance of this developmental period, relatively little is known about the emergence and development of response inhibition, a form of inhibitory control, and the associated neural substrates across this key transition. Using functional near-infrared spectroscopy (fNIRS), an optical imaging technique suitable for imaging the developing brain, and an age-appropriate response inhibition task, we investigated the brain regions associated with response inhibition in 16-month-old toddlers. This pre-registered study extends our previous work with 10-month-old infants (Fiske et al., 2022) as it follows the same cohort of participants, now at 16 months of age. Whilst our previous work demonstrated that 10-month-old infants recruited right-lateralised regions of the PFC and parietal cortex when inhibition was required, the current study suggests that by 16 months, toddlers recruit the left superior parietal gyrus, the right inferior frontal gyrus, and bilateral regions of the dorsolateral PFC and orbital frontal cortex. Although there was no longitudinal change in response inhibition performance, more widespread, bilateral regions of the PFC were recruited during response inhibition at 16 months compared with 10 months. We acknowledge the need for replication of these results. Nevertheless, our findings suggest that the transition from infancy to toddlerhood may constitute an important period of reorganisation of the PFC that might support the development of early inhibitory control processes.

## 1 Introduction

Inhibitory control is a core executive function (EF) skill that enables the exertion of control over thoughts, actions, and behaviours. This skill has been reliably measured already in the first year (Bell & Fox, 1992;Diamond, 1985;Fiske et al., 2022;Hendry et al., 2021;Holmboe et al., 2008) and continues to show substantial progression across the first 3 years of life (Hendry et al., 2016;Holmboe et al., 2021). Importantly, early inhibitory control is predictive of several outcomes later in life, such as self-regulation (Nigg, 2017), academic skills (Bull & Scerif, 2001;St Clair-Thompson & Gathercole, 2006), adolescent EF (Friedman et al., 2011), and health and well-being in adolescence and adulthood (Anzman-Frasca et al., 2015;Moffitt et al., 2011). Despite this, knowledge about inhibitory control in the transition from infancy to early toddlerhood is significantly lacking (“the toddler data desert”;Kerr-German et al., 2023). As such, research is needed that addresses how inhibitory control skills emerge and develop across infancy and toddlerhood.

When examining the development of early EFs in the first years of life,Garon et al. (2008)proposed a hierarchical model, such that simple component skills early in infancy become the building blocks for more complex EF skills that develop with age (Devine et al., 2019;Johansson et al., 2016). Research has evidenced that simple inhibition skills emerge early in infancy and can be reliably measured in the second half of the first year (Clearfield et al., 2006;Diamond, 1985;Holmboe et al., 2008,^2018^), and more sophisticated inhibitory skills continue to develop across early childhood (Cuevas & Bell, 2014;Hendry et al., 2016;Moilanen et al., 2010). Whilst EF skills (inhibitory control, working memory, and cognitive flexibility;Diamond, 2013) may appear fractionated in infancy and early toddlerhood (i.e., different EF measures show limited correlations;Miller et al., 2023), by late toddlerhood, coherence between measures emerges with components often loading onto a single EF factor (Broomell & Bell, 2022;Wiebe et al., 2011).

Underpinning the substantial inhibitory control development in the early years of life is the structural and functional maturation of the prefrontal cortex (PFC), an area of the brain that has been consistently associated with higher-order executive processes (Diamond, 2002;Fiske & Holmboe, 2019;Klingberg et al., 2002). Similarly, there is increasing evidence to support the role of the parietal cortex (Cai et al., 2016;Kolodny et al., 2017;Tamnes et al., 2013), as well as a frontoparietal network (Aron et al., 2007;Niendam et al., 2012), as key neural substrates of inhibitory control. However, until relatively recently, it has been difficult to examine the neural correlates of inhibitory control in developmental populations. This is likely due to methodological limitations, such as a lack of age-appropriate tasks (Holmboe et al., 2021) and the limited availability of suitable neuroimaging techniques for this early developmental population (Lloyd-Fox et al., 2010).

Electroencephalography (EEG) studies have contributed significant knowledge about the broad involvement of frontal and parietal regions (Bell & Wolfe, 2007;Bell et al., 2007;Broomell et al., 2021;Cuevas et al., 2012), but have been unable to localise activation within these cortical regions due to the spatial limitations of EEG. Since better spatial resolution can be achieved with functional near-infrared spectroscopy (fNIRS), it is now possible to functionally localise neural activation to specific brain regions in developmental populations (Gervain et al., 2023;Lloyd-Fox et al., 2010). Indeed, previous research has used fNIRS to investigate the functional neural substrates of response inhibition in infants (Fiske et al., 2022), toddlers (Kerr-German et al., 2022), pre-schoolers (Kerr-German & Buss, 2020;Monden et al., 2015;Moriguchi et al., 2018), and young children (Mehnert et al., 2013;Zhou et al., 2022). Overall, evidence from these fNIRS studies consistently demonstrates the involvement of the prefrontal and parietal cortices in response inhibition tasks across infancy and early childhood, although evidence from the second year of life is currently limited.

Despite the growing body of fNIRS studies investigating inhibitory control across development, very few studies have examined the potential stability or change in the neural correlates of response inhibition*longitudinally*during the first years of life. Yet, the transition from late infancy to toddlerhood is a fundamental developmental period where many changes occur across multiple domains. For example, neuroscientific research into early childhood structural brain development has demonstrated that the early years of life are a fundamental time for cortical maturation (Croteau-Chonka et al., 2016;Gilmore et al., 2018;Remer et al., 2017), and there is evidence to suggest that the PFC may also be undergoing a transitional period of maturation across infancy and early childhood (Herschkowitz, 2000). It is interesting to note that structural changes in the PFC across infancy and early childhood appear to coincide with a period of rapid improvement in EF performance (Diamond, 1985;Diamond & Doar, 1989;Diamond & Goldman-Rakic, 1989;Fiske & Holmboe, 2019).

It would have significant implications for the field to have a picture of the developmental trajectory of inhibitory control from its emergence in infancy and across toddlerhood, as well as an understanding of the change in the functional neural substrates that underpin this development. It is of great importance to understand the mechanisms by which the frontal cortex and other areas in the executive function network mature early in infancy, and how they support the rapid development of cognitive skills such as inhibitory control in the first years of life. By examining neural development in early life, it may be possible to identify and understand potential mechanisms that relate to cognitive development at an earlier age than is possible with behavioural studies.

### 1.1 The current study

The overarching aim of the current study was to investigate the functional neural correlates of response inhibition in 16-month-old toddlers. The transition from infancy to toddlerhood marks an important period for the development of early EFs (Devine et al., 2019;Hendry et al., 2016). For example, coherence across different EF tasks and longitudinal stability in performance start to emerge in toddlerhood (Broomell & Bell, 2022;Hendry et al., 2021;Holmboe et al., 2021;Miller et al., 2023). However, there is currently a significant lack of knowledge about both the behavioural development and associated neural correlates of inhibitory control in the second year of life. Whilst our previous study (Fiske et al., 2022) investigated this in 10-month-old infants, the current paper reports on the same cohort of participants, now at 16 months of age, to contribute new knowledge about the neural correlates of response inhibition across the transition to early toddlerhood. Overall, this study aimed to investigate the behavioural development of response inhibition, and the change in associated neural correlates, from 10 to 16 months.

Participants completed the Early Childhood Inhibitory Touchscreen Task (ECITT;Fiske et al., 2022;Hendry et al., 2021;Holmboe et al., 2021) whilst their brain activity was measured using fNIRS across the prefrontal and parietal cortices. The ECITT has been shown to be a reliable measure of response inhibition in infants as young as 10 months of age (Fiske et al., 2022;Hendry et al., 2021;Lui et al., 2021), in toddlers aged 18–30 months (Holmboe et al., 2021), and across the lifespan (Holmboe et al., 2021). This task encourages the development of a prepotent (well-learned) motor response by presenting a target stimulus (smiley face icon) on one side of the touchscreen on 75% of trials (prepotent trials), and rewarding correct responses with a short, animated cartoon. As the target appears on the opposite side of the screen on 25% of trials (inhibitory trials), participants are required to occasionally inhibit their prepotent response by touching the other side of the screen. A blocked version of the ECITT has recently been developed for fNIRS studies (Fiske et al., 2022). Neural responses in blocks of trials when inhibition is required (experimental blocks; mixed prepotent and inhibitory trials) are contrasted with blocks of trials where only prepotent responses are required (control blocks; prepotent trials only).

Since the current study involved 16-month-old toddlers who were part of a longitudinal study (data from this sample of participants at 10 months of age is reported inFiske et al. (2022)), we were able to examine longitudinal change across the transition from late infancy and into toddlerhood, using the same experimental paradigm. As such, the results of this study contribute new knowledge about how the maturation of frontal and parietal cortices may support the development of response inhibition skills from the first to the second years of life.

Our research questions, hypotheses and analysis plans were pre-registered using the Secondary Data Preregistration template on the Open Science Framework website (https://osf.io/hpb4s). Our main hypotheses are outlined below. Based on evidence from previous studies that have used the ECITT with infants and toddlers (Fiske et al., 2022;Hendry et al., 2021;Holmboe et al., 2021), it was predicted that toddlers would be significantly more accurate on prepotent trials than on inhibitory trials and would respond significantly faster on correct prepotent trials than on correct inhibitory trials. Considering the fNIRS data, it was predicted that the same channels that were significantly more active when inhibition was required at 10 months (Fiske et al., 2022) would also be recruited when inhibition was required at 16 months (channels covering the right parietal cortex, the right dorsolateral PFC (DLPFC), and the right orbital frontal cortex (OFC)). We also pre-registered that we would conduct exploratory analyses to examine whether any other channels showed significantly more activation when inhibition was required (compared with when inhibition was not required). Similarly, it was pre-registered that we would conduct exploratory analyses to investigate potential associations between individual differences in neural activation and performance on the ECITT, and also to investigate the longitudinal change in the neural correlates of response inhibition from 10 to 16 months.

## 2 Method

### 2.1 Participants and data exclusions

Participants were 103 16-month-old toddlers who were recruited to the Oxford Early Executive Functions (OEEF) study shortly before they were 10 months of age. The OEEF study is a larger longitudinal study that aims to examine the development of early executive functions in the first years of life (University of Oxford Central University Research Ethics Committee: R57972/RE010). The study assessment points (10, 16, 24, and 30 months) were evenly spaced (6–8 months apart) across the first 2.5 years to ensure that we would observe developmental change between the ages. The choice of the second age point (16 months) was to some extent determined by practical considerations (making sure that age points were reasonably spaced apart) but also the lack of existing data during the early toddlerhood period. Written informed consent was obtained from the caregivers of all participants before being included in the study. Families received a £20 online shopping voucher and a Babylab branded gift for their participation.

The data reported in the current study were collected at the second assessment point of the OEEF study, when participants were 16 months of age. Participants were invited to attend two testing sessions at the Oxford University Babylab (~1.5 hours each) where toddlers completed a battery of age-appropriate executive function tasks. A total of 103 participants (*N*= 55 male toddlers) attended their first in-person test session, and 98 participants (*N*= 52 male toddlers) returned for the second test session approximately 1 week later. As per the OEEF study inclusion criteria, one female participant was excluded from the study due to her low birth weight, and three male participants were excluded due to birth complications leading to health-related concerns.

In the first test session, participants completed a “behavioural” version of the ECITT (this version of the task is described inHendry et al. (2021)) and in the second test session participants completed a “blocked” version of the ECITT alongside fNIRS. These two versions of ECITT (“behavioural” and “blocked”) were used to establish convergence in behavioural findings between a version of the task that intermixes trial types (“behavioural”) and one that is more appropriate for fNIRS data collection (“blocked”). The blocked design allows for the comparison of neural activation in block types with an inhibitory demand (experimental blocks) and without an inhibitory demand (control blocks). SeeSection 2.2.2below for more details about the two versions of the ECITT.

**Fig. 1.**
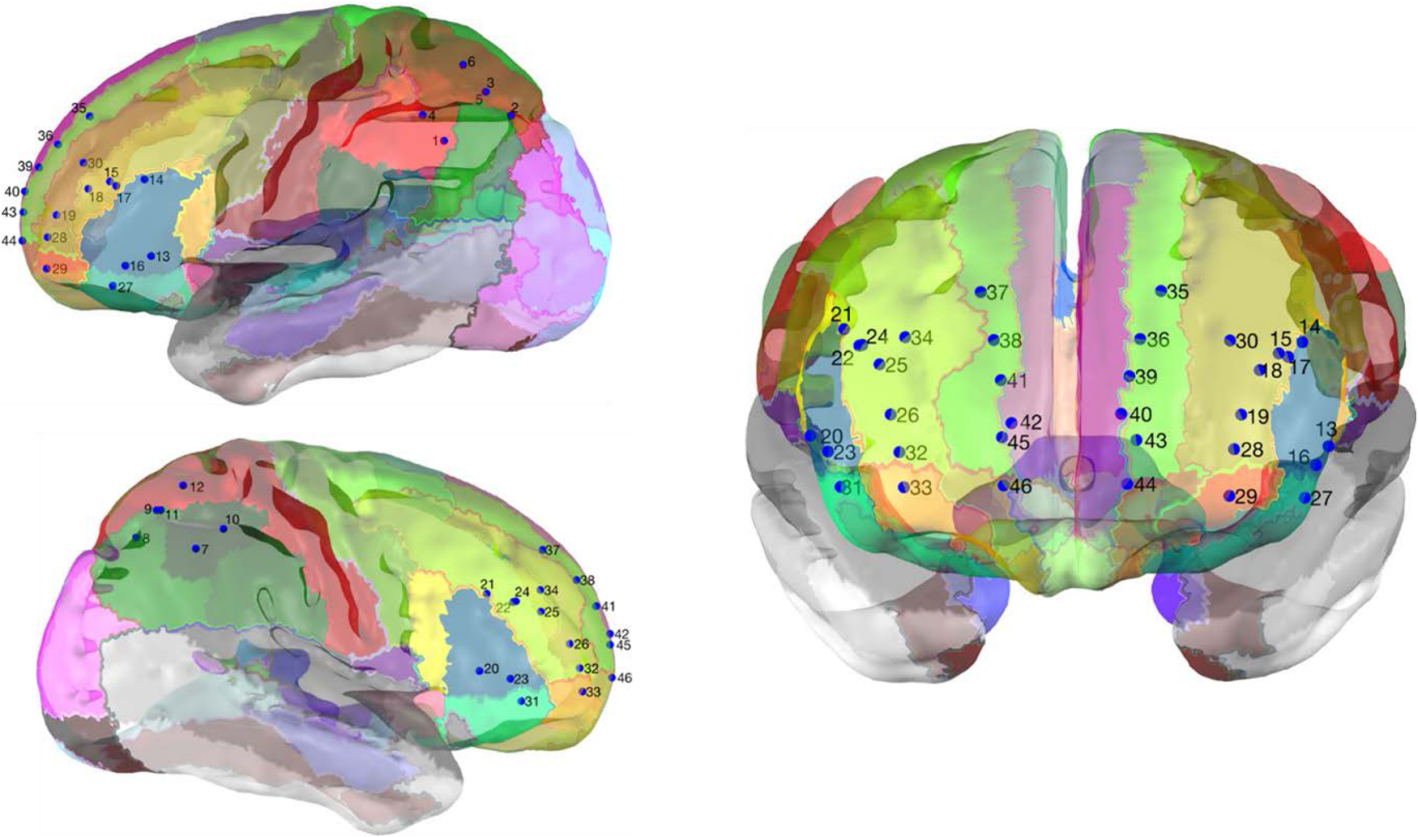
fNIRS probe channel positions.*Note.*Blue points represent channel positions on the cortex and numbers refer to the channel numbers. SeeSupplementary Materials 2.1for a 2D channel map, andSupplementary Materials 2.4for a table providing the anatomical location of each channel. The fNIRS probe covers the bilateral prefrontal cortex and the bilateral area around the intraparietal sulcus. Head model is based on averaged structural MRI data of a 12-month-old cohort of infants (Shi et al., 2011) and was scaled to the group mean head circumference measurement of participants in this study with useable fNIRS data. The positions of sources and detectors were registered virtually to the scalp surface of the head model using the HomER2 spring relaxation mechanism (Aasted et al., 2015). MATLAB figures of the channel positions on the cortex are available to viewhere.

It is worth noting that the participant sample at 16 months was limited in that data collection had to stop prematurely because of the COVID-19 pandemic. As such, some participants who were tested at 10 months were unable to return for their session at 16 months (*N*~ 66), and so the sample size was smaller than originally planned at this assessment point. To maximise statistical power at this time point, 16-month data from an additional 25 participants are included in the current sample. These participants were recruited as part of pilot test sessions for the longitudinal study and were assessed under the same administration protocol as participants in the longitudinal study at 16 months. Many of these pilot participants contributed behavioural ECITT data when they were 10 months old (reported inHendry et al. (2021)), however, did not contribute blocked ECITT and/or fNIRS data to our previous publication (Fiske et al., 2022) because the blocked ECITT task was completed under a different administration protocol at this point in the piloting process (i.e., using an early version of the task, when the pilot participants were 10 months old). At 16 months, the ECITT protocol for both versions of the task was identical across the two samples (seehere). Demographic information for the full sample is reported inSupplementary Materials 1, and a visual breakdown of the different samples and administration protocols is presented inSupplementary Figure 1.

After task-level exclusions (seeTable 1), the final sample with behavioural ECITT data (Session 1) consisted of 77 participants, and the final blocked ECITT sample (Session 2) consisted of 81 participants. A total of 67 toddlers had valid ECITT data from both sessions. Of the 81 participants with valid blocked ECITT data, a sub-sample of 43 participants also contributed valid fNIRS data.

**Table 1.** Data exclusions.

Session 1 Behavioural data (behavioural ECITT)	*N*
Less than 16 trials completed (including at least 2 valid inhibitory trials)	2
Less than 60% accuracy on prepotent trials	19
Software failure: data did not save	1
Total excluded	22
Percentage excluded	22.22%
Total included	77

Session 2 Behavioural data (blocked ECITT)	*N*
Did not complete task	-
Less than 2 blocks of each type completed (4 blocks total)	8
Less than 60% accuracy on prepotent trials	4
Video of session was not recorded	1
Total excluded	13
Percentage excluded	13.83%
Total included	81

fNIRS data	*N*
Data file was lost	-
Refused to wear fNIRS cap	8
Did not complete at least 3 blocks of each type (6 blocks total)	3
fNIRS cap was removed before the minimum number of blocks had been completed	5
fNIRS cap placement was invalid	16
Did not have at least 3 valid blocks of each type (6 valid blocks total)	4
Did not meet pre-processing data quality criteria	2
Total excluded	38
Percentage excluded	53.08%
Total included	43

*Note.*The percentage excluded for the fNIRS data refers to the percentage of the 81 participants with usable blocked ECITT data.

### 2.2 Apparatus and stimuli

#### 2.2.1 Gowerlabs NTS fNIRS system

The Gowerlabs NTS continuous wave fNIRS system was used to collect brain data from participants. The fNIRS probe consisted of 32 optical sensors forming 46 channels that bilaterally covered the PFC and the area around the intraparietal sulcus (Fig. 1) and seeSupplementary Materials 2.1for a 2D channel map. A full description of the fNIRS system and probe design is available inFiske et al. (2022).

The fNIRS cap was fitted to the participant’s head so that the front of the cap sat just above the eyebrows, and the optode anchored to FpZ was positioned centrally between the eyebrows. The headgear placement was standardised across participants (as much as possible) to ensure that the optical sensors were covering the brain areas they were designed to cover. At the start of the fNIRS test session, photographs or videos were taken as a record of how the fNIRS cap was positioned on the child’s head. If there was significant cap movement during the session, additional photographs/videos were recorded at the end of the session to document the movement of the cap on the head. The validity of the headgear placement was assessed by two independent coders who viewed the photographs and videos and rated the headgear placement using a simple coding scheme. Coding discrepancies were reviewed by a third coder who made an independent judgement using the same coding scheme. The coders reviewed discrepancies together and agreed on a final decision based on the majority validity judgement. Headgear placement was coded as “good” if the edge of the cap was sat straight on the head and on the eyebrows or just very slightly above the eyebrows, with the central optode (FpZ) in the centre of the forehead, directly above the nose and level with the eyebrows. Headgear placement was coded as “acceptable” if the cap was sitting a little high and/or a little lop-sided on the head with the central optode (FpZ) slightly off-centre but looked acceptable overall. Headgear placement was coded as “poor” if the cap was sitting very high (halfway up the forehead or higher) and/or was not on straight at all, with the central optode being nowhere near the centre of the forehead (more than halfway over the eyebrow, from the middle of the eyebrow and beyond). Headgear placement that was rated “poor” was deemed invalid and the participant’s fNIRS data were not included in the analyses.

#### 2.2.2 Early Childhood Inhibitory Touchscreen Task (ECITT)

Two versions of the ECITT were used in the OEEF study. The “behavioural ECITT” (as described inHendry et al. (2021)) was administered in Session 1 and consisted of a single block of 32 continuous trials (75% prepotent, 25% inhibitory), whereas the “blocked ECITT” (as described inFiske et al. (2022)) was administered in Session 2 alongside fNIRS. The blocked ECITT consisted of*control blocks*(six prepotent trials; no inhibitory demand) and*experimental blocks*(three prepotent and three inhibitory trials) that were separated by a baseline block (moving abstract shapes accompanied by calm music, jittered in duration from 12 to 17 s). Thus, the allocation of 75% prepotent and 25% inhibitory trials was ensured across blocks. In the blocked ECITT, trials were randomised with the constraints that the target (smiley) was always presented in the prepotent location on the first trial of a block and the target could not consecutively appear in the same location more than twice. Please seeFigure 2below for an example of the block sequence and seeFiske et al. (2022)for a full description of the blocked ECITT. A copy of the administration protocol can be foundhere. Details about how to obtain the code to administer the ECITT are provided inHolmboe et al. (2021).

**Fig. 2.**
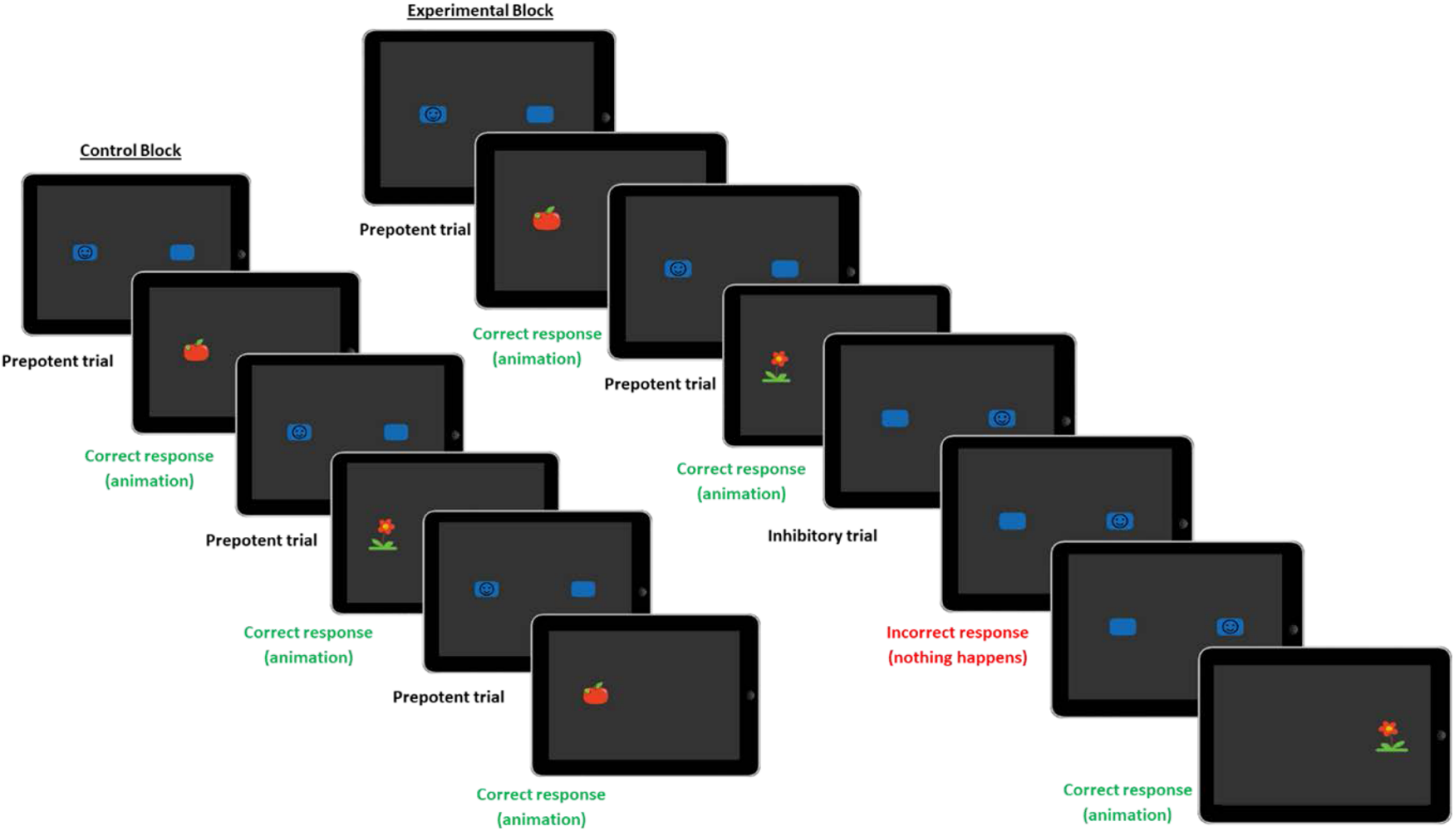
The blocked ECITT.

The number of blocks completed by participants with valid fNIRS data in the current study ranged from 3 to 8 of each block type (Control:*M*= 5.22, Experimental:*M*= 5.04), with a mean duration of 35 s (*SD*= 9.8 s) for control blocks and 42 s (*SD*= 11.7 s) for experimental blocks. Overall, participants with valid fNIRS data completed between 6 and 15 blocks of trials (*M*= 10.27), which is equivalent to 36–90 individual trials.

### 2.3 Procedure

For both test sessions, participants were seated on their caregiver’s lap and positioned at a table, adjacent to the experimenter. In Session 1, participants completed the behavioural ECITT, which began with a single demonstration trial followed by at least one practice trial (a single target centrally presented) before the first (of 32) experimental trial was presented. The task was continued until participants had completed 32 trials or disengaged or became fussy (in which case the task was stopped). In Session 2, participants wore the fNIRS cap whilst they completed the blocked ECITT. There was no demonstration or practice trials. As the blocked ECITT has no stopping point, the experimenter stopped the task when the participant became fussy or distracted, although participants were encouraged to complete at least three blocks of each type to ensure enough usable fNIRS data had been collected.

In both versions of the task, the first trial was always cued by the experimenter with the instruction “can you touch the happy face?”. As such, the first trial was always excluded from analysis. Verbal encouragement was given when necessary to increase engagement but was kept to a minimum during the fNIRS session. The experimenter removed the iPad from the participant’s reach (although still within their sight) during the animations that occurred after each trial and during baseline blocks to minimise the occurrence of premature or accidental responses (which have to be excluded). Please see our OSF project (https://osf.io/cpnk9) for the full administration protocols for both versions of the ECITT.

### 2.4 Data processing

#### 2.4.1 Behavioural data processing

Results of the inter-coder reliability achieved for the accuracy and validity coding for both versions of the ECITT, and for the baseline looking behaviour coding, are reported inTable 2; excellent inter-coder reliability was achieved for all measures. Coders were also trained to identify trials in which the reaction time needed correcting (e.g., because the child’s first response was not detected by the iPad) and to use a video player with a frame counter to make the necessary timing corrections. Videos of the session were recorded at a frame rate of 33 ms per frame, and, therefore, the accuracy of the corrected response times is limited to this margin of error. Also note that during the behavioural data processing, two participant sub-samples were formed. These consisted of those participants who contributed valid fNIRS data and valid blocked ECITT data (*N*= 43) and those who contributed valid blocked ECITT data only (*N*= 38). Our behavioural ECITT coding protocol can be foundhereand our baseline looking behaviour coding protocol can be foundhere.

**Table 2.** ECITT inter-coder reliability.

	No of coders	No of videos ( *N* of coding instances)	Test	Accuracy	Validity
Behavioural ECITT	3	20 (644)	Fleiss’ kappa	.943	.840
Blocked ECITT	2	20 (1234)	Cohen’s kappa	.953	.882
Baseline looking behaviour	2	20 (1064)	Cohen’s kappa	-	.890
Reaction time corrections— behavioural ECITT	2	20 (94)	Intraclass correlation	.974	-
Reaction time corrections— blocked ECITT	2	20 (98)	Intraclass correlation	.989	-

*Note.*Three coders were trained on the behavioural ECITT task, two of whom were also trained in the reaction time correction coding for this task. Two coders were trained on the “blocked ECITT” task (including the reaction time correction and baseline looking behaviour). All coders attained excellent intercoder reliability (Kappa/ICC > .80).

#### 2.4.2 fNIRS data processing

The Gowerlabs NTS system measured a raw intensity signal (two wavelengths: 780 and 850 nm) that underwent several transformations during pre-processing in HomER2 (Huppert et al., 2009), seeSupplementary Materials 2.2. for a full description of the preprocessing pipeline. Participants with more than two-third of channels excluded following the pruning of poor-quality channels were not included in further analysis. Trial blocks were coded as invalid (and then excluded) if the duration of the block was longer than the mean duration + 2*SD*for each block type. As such, control blocks were excluded if they exceeded 54 s, and experimental blocks were excluded if they exceeded 65 s. The mean duration of control blocks was 35 s (*SD*= 9.8 s) and the mean duration of experimental blocks was 42 s (*SD*= 11.7 s).

Oxygenated haemoglobin (HbO_2_) and deoxygenated haemoglobin (HHb) concentration change data from each channel were block averaged over a period of 37 s (containing 2 s of data from valid baseline blocks and 35 s of data from valid task blocks). The 35-s analysis window was selected based on the mean duration of control blocks (stated above). Baseline concentration change data (2 s preceding the next block) were subtracted from the average haemoglobin concentrations in the 35-s experimental window and the baseline-corrected data were divided into seven 5-s time bins to equally distribute time bins across the time window. Since the task is response-contingent, the duration of each block varied both between and within participants. As such, the block time course was limited to 35 s to ensure that all time windows contained only task block data for all participants, rather than a mixture of task block data for some participants and baseline data for others.

### 2.5 Analysis approach

**Fig. 3.**
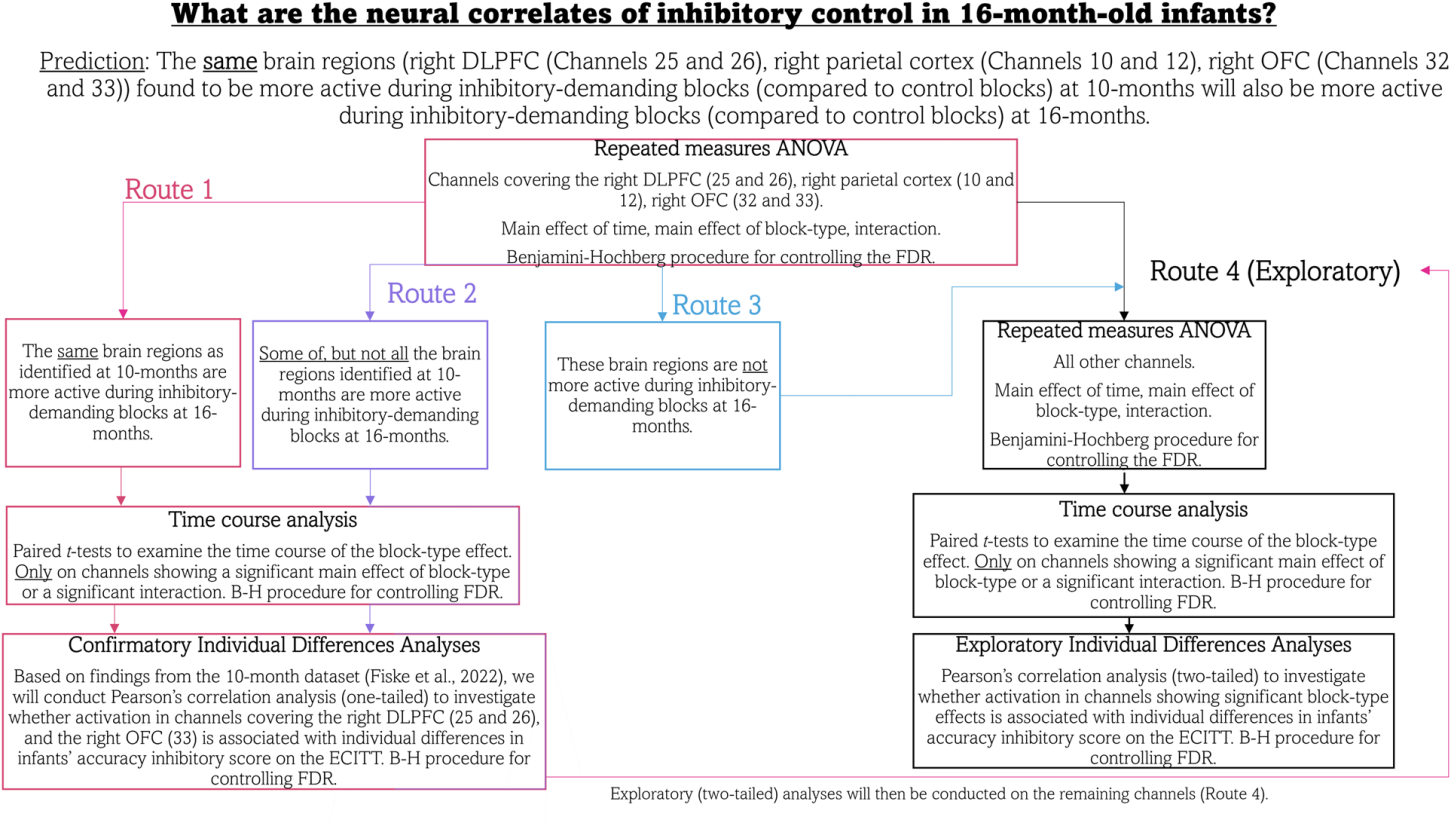
Pre-registered analysis plan for fNIRS data.*Note.*This flow chart diagram is from our pre-registration (https://osf.io/hpb4s) and illustrates the analysis plan for the fNIRS data. Each of the four routes depicts the analysis approach we would take based on the outcome of the repeated measures ANOVA. Routes 1–3 related to our hypothesis that the same brain regions (as found at 10 months) would also be showing significant condition effects at 16 months, and as such were confirmatory. Note that the route taken would depend on the outcome of the repeated measures ANOVA, and that these analyses would only be conducted using data from the pre-registered channels. Route 4 was an exploratory analysis route whereby we would conduct the analyses on all remaining channels in the fNIRS probe.

The hypotheses, variables, and analysis plan were defined and pre-registered prior to analysis; for full details, please seehttps://osf.io/hpb4s.Figure 3displays a flow chart overview of the pre-registered analysis plan for the fNIRS data. Prior to submitting the pre-registration, the anatomical labels of brain regions covered by channels in the fNIRS probe at 16 months were generated by Dr. Liam Collins-Jones to support the predictions of which channels would show inhibition-related activation at 16 months. This was an important step considering changes in head size from 10 to 16 months of age. The head modelling and channel localisation process were the same as inFiske et al. (2022)and can be found inSupplementary Materials 2.3. SeeSupplementary Materials 2.4for the anatomical labels of all channels in the probe.

In this work, two analysis approaches were taken. First, our primary analyses were conducted at the channel level (as outlined inFig. 3below). These were repeated measures ANOVAs to investigate which channels were showing a significant block-type effect, that is, a significantly greater increase in HbO_2_/decrease in HHb in experimental blocks (where there is an inhibitory demand) than in control blocks (no inhibitory demand). Paired*t*-tests were also conducted on channels showing significant block-type effects in order to investigate the time course of the significant effect. Finally, correlational analyses were conducted to investigate associations between activation and individual differences in performance. As such, our discussion of the results will be in the channel space.

Our secondary analyses were conducted in image space, where we took an image reconstruction approach to enable us to visualise the significant block-type effects as projected onto the cortex. The output of the image reconstruction approach is an image of changes for each time point during the block-averaged time window. These values at each node are then compared with a short baseline period using a two-sample*t*-test to produce the reconstructed image. This image space analysis of the fNIRS data was conducted as it accounts for light propagation through the different layers of the head, as opposed to other forms of visualisation which do not account for anatomical differences across the head, such as interpolating*t*-stat values projected onto the cortex. However, the lack of overlapping channels in our fNIRS array (channels that sample overlapping volumes of tissue; seeSupplementary Fig. 1) is a limitation for this approach. Whilst image reconstruction approaches have been successful with low-density fNIRS arrays (Collins-Jones et al., 2021), it is worth noting that image reconstruction methods are better suited to higher density arrays with many overlapping channels (Eggebrecht et al., 2014;White & Culver, 2010). Whilst it is possible to extract time course HRFs from a particular region in the image space, given the low density of the fNIRS array, for our primary analyses we chose to go with pipelines to preprocess/process the data in the channel space which have been used extensively in previous infant fNIRS research (Blanco et al., 2023;Frijia et al., 2021;Issard & Gervain, 2018;Lloyd-Fox et al., 2019).

#### 2.5.1 Variables

The primary behavioural variables in the current study are mean accuracy and median reaction time for the two trial types (prepotent, inhibitory). The mean accuracy variables were calculated for each participant as the mean accuracy across all valid trials and was scored from 0 (all incorrect) to 1 (all correct). The median reaction time variables were calculated for each participant as the median reaction time across all valid and correct trials. Additionally, an accuracy inhibitory score was used as an index of response inhibition performance such that better response inhibition was indicated by a higher accuracy inhibitory score.

Following pre-processing (described inSection 2.4.2. above), two primary fNIRS variables were generated for use in group-level analyses. An average HbO_2_concentration variable and an average HHb concentration variable were generated for each participant with valid fNIRS data (*N*= 43), for each time bin (*N*= 7) and for each channel (*N*~46, although actual number varies per participant due to the exclusion of poor-quality channels). These data were entered into repeated measures ANOVAs to identify channels showing significant block-type effects (e.g., significantly greater HbO_2_concentration increase or significantly greater HHb decrease in experimental blocks compared with control blocks). For the purposes of individual differences analyses, we calculated the difference between HbO_2_concentrations in experimental and control blocks (experimental minus control; “HbO_2_difference”), and the difference between HHb concentrations in control and experimental blocks (control minus experimental; “HHb difference”) and created an average of this difference measure across channels identified in group-level analyses as showing significant block-type effects. Please seeSupplementary Materials 3for a full description of the behavioural and fNIRS variables used in this study.

#### 2.5.2 Statistical analysis

All statistical analyses were conducted in SPSS version 29. All variables were tested against the parametric test assumptions (Supplementary Materials 4), and when violated, appropriate non-parametric equivalents were used (reported inSupplementary Materials 5). Greenhouse-Geisser corrected degrees of freedom and significance values were used because the channel-level fNIRS data did not meet the sphericity assumption required for repeated measures ANOVA. The procedure for controlling the false discovery rate (FDR;Benjamini & Hochberg, 1995) was used where multiple tests were conducted. CIs (95%) were calculated on 1000 bootstrap samples. Note that behavioural ECITT data from the 25 pilot participants will not be included in analyses that examine trial-type effects, as these data have already been reported inHendry et al. (2021).

## 3 Results

### 3.1 Behavioural results

In line with our predictions and replicating previous work (Fiske et al., 2022;Hendry et al., 2021;Holmboe et al., 2021), results demonstrated that mean accuracy was significantly higher, and median reaction time was significantly faster on prepotent trials than on inhibitory trials on both versions of the ECITT (reported inTable 3below).

**Table 3.** Performance on the ECITT at 16 months.

Accuracy	*N*	Inhibitory	Prepotent	Test statistic	*p*	Effect size
Session 1	61	.486 (.340)	.921 (.082)	*t* (60) = -9.304	<.001	*d* = -1.191
Session 2	81	.536 (.324)	.924 (.088)	*F* (1, 79) = 102.745	<.001	ηp ^2^ = .565

Reaction time (ms)	*N*	Inhibitory	Prepotent	Test statistic	*p*	Effect size
Session 1	53	1627 (514)	1457 (356)	*t* (52) = 2.604	.006	*d* = .358
Session 2	77	1454 (418)	1280 (216)	*F* (1, 75) = 19.435	<.001	ηp ^2^ = .206

*Note.*Mean (*SD*) is reported for the accuracy variables and median (*SD*) is reported for the reaction time variables. The*N*’s for reaction time data are smaller than for accuracy because no reaction time data could be generated if participants did not have at least one valid correct inhibitory trial. Note that behavioural ECITT (Session 1) data from the 25 pilot participants were not included in these analyses, as these data have already been reported inHendry et al. (2021). An ANOVA was used for Session 2 data because we also include “sub-sample” in the ANOVA that tests whether there was a significant difference between trial types and sub-samples (seeSection 3.1.2. for more details).

To examine behavioural development (mean accuracy) from 10 to 16 months, two separate 2 × 2 (trial type, age) repeated measures linear mixed models were conducted (one model for each version of the ECITT). Results (reported inSupplementary Materials 5) confirmed that infants were significantly more accurate on prepotent than on inhibitory trials in both sessions/versions. However, there was no significant main effect of age, nor a significant trial type × age interaction; seeFigure 4. These results are in line with the prediction (based on prior results) that there would be no significant performance improvement from 10 to 16 months. There was also no significant association between the accuracy inhibitory score at 10 and 16 months on the behavioural ECITT (Session 1):*r*(63) = .144,*p*= .126,*CI*= [-.128, .385], or on the blocked ECITT (Session 2):*r*(60) = -.044,*p*= .368,*CI*= [-.275, .196].

**Fig. 4.**
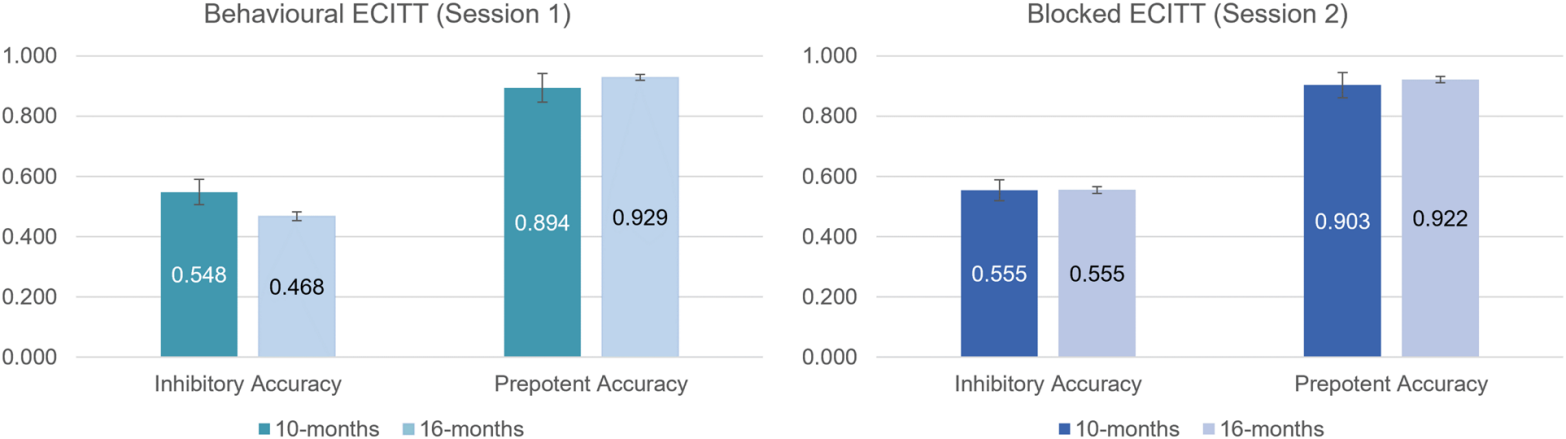
Behavioural development of response inhibition from 10 to 16 months.*Note.*The reported values represent the mean and error bars represent the standard error.*N*= 52 for behavioural ECITT (Session 1, excluding*N*= 13 pilot participants), and*N*= 62 for blocked ECITT (Session 2).

#### 3.1.1 Test–retest reliability and between-session consistency at 16 months

Results of correlational analyses indicated moderate test**–**retest reliability for the mean inhibitory accuracy (*r = *.572,*p*<.001), accuracy inhibitory score (*r = *.536,*p*< .001), and median prepotent reaction time (*r = *.397,*p*= .001) variables, as predicted. However contrary to expectations, there was no significant correlation across sessions for median inhibitory reaction time and mean prepotent accuracy. Results of between-session consistency analyses (2 × 2 repeated measures ANOVAs; trial type, session) revealed that infants were significantly more accurate (*p*= .003, ηp^2^= .128), and responded significantly faster (*p*= <.001, ηp^2^= .195) on the blocked ECITT in Session 2 than on the behavioural ECITT in Session 1, contrary to predictions. SeeSupplementary Materials 5for full results.

#### 3.1.2 Behavioural performance by sub-sample

To test whether performance significantly differed by trial type (inhibitory, prepotent) or between sub-samples (those with valid fNIRS data (*N = *43) and those with only valid ECITT data (*N*= 38)), two separate 2 × 2 mixed ANOVAs were conducted on the accuracy and reaction time data from the blocked ECITT (Session 2); estimated marginal means are reported inSupplementary Materials 5. Results demonstrated that there was no significant trial × sub-sample interaction for either the accuracy (*F*(1, 79) = 1.412,*p*= .238, ηp^2 = ^.018) or the reaction time (*F*(1, 75) = .221,*p*= .640, ηp^2 = ^.003) variables. There was no significant main effect of sub-sample for the accuracy data (*F*(1, 79) = 1.195,*p*= .278, ηp^2 = ^.015), however, there was a significant effect of sub-sample for the reaction time data (*F*(1, 75) = 6.456,*p*= .013, ηp^2 = ^.079). This suggests that participants who contributed valid fNIRS data responded significantly faster than those without valid fNIRS data. Although the significant difference in overall median reaction time between sub-samples was unexpected, the predicted trial-type effects were fully confirmed, were consistent across sub-samples, and mostly had medium to large effect sizes.

### 3.2 fNIRS group-level results

The purpose of these analyses was to identify channels demonstrating a significant haemodynamic response (increase in HbO_2_and/or decrease in HHb from baseline) that significantly differed between block types (control, experimental). For example, we expected to observe channels showing a significantly larger increase in HbO_2_and/or larger decrease in HHb during experimental blocks (inhibitory demanding) than in control blocks (no inhibitory demand). In the pre-registration, we hypothesised that six channels (found to be showing significant block-type effects at 10 months) would also show significant block-type effects at 16 months (Route 1 of the analysis flow chart inFig. 3). These channels were Channels 10 and 12 (overlying the right parietal cortex), Channels 25 and 26 (right DLPFC), and Channels 32 and 33 (right OFC). Following pre-processing, Channels 10 and 12 (right parietal) had to be excluded from the confirmatory group-level analysis because less than 70% of participants contributed data to these channels. We believe that the poor signal quality in these channels was due to poor optode coupling, likely as a result of the position of the channels where hair is at its thickest.

Repeated measures ANOVAs were separately conducted on data from the remaining four channels (Route 1 of the analysis flow chart inFig. 3). The results revealed that all four channels demonstrated a significant main effect of time (i.e., the haemoglobin concentration changed over time from baseline; seeSupplementary Materials 6). Two channels overlying the right DLPFC and one overlying the right OFC showed a significant main effect of time in both the HbO_2_and HHb signals, whereas one channel overlying the right OFC only showed a significant main effect of time in the HHb signal. Following correction for the FDR, all channels retained significance for the main effect of time. A significant main effect of block type was observed in the HHb signal for one channel overlying the right OFC (Table 4), suggesting that the HHb concentration in this channel was differentiated by block type. A significant time × block-type interaction was found in the HbO_2_signal for a channel overlying the right DLPFC (Table 4), suggesting that there was a significant difference in HbO_2_concentration between experimental and control blocks, and that this difference was dependent on the time bin. None of the effects survived correction for the FDR (eight comparisons; four channels × two chromophores).

**Table 4.** Results of repeated measures ANOVA: channels showing significant effects.

Location	Hemisphere	Channel	Signal	Statistic
Superior parietal gyrus	Left	6	HHb	*F* (1, 38) = 5.530, *p* = .024, ηp ^2^ = .127
IFG	Right	23	HHb	*F* (1, 42) = 4.191, *p* = .047, ηp ^2^ = .091
DLPFC	Left	28	HHb	*F* (1, 41) = 8.495, *p* = .006, ηp ^2^ = .172
	Right	** 26 ^a^**	HbO _2_	*F* (3.557, 128.065) = 2.595, *p* = .046, ηp ^2^ = .067
OFC	Left	29	HHb	*F* (1, 41) = 4.158, *p* = .048, ηp ^2^ = .092
	Right	** 33 ^a^**	HHb	*F* (1, 42) = 4.370, *p* = .043, ηp ^2^ = .094

*Note.*Bolded channels also showed significant effects in the 10-month study, and we had pre-registered hypotheses predicting effects in these channels. Note that the significant effect found in Channel 26 was a time × block-type interaction, whereas all other channels showed a significant main effect of block type.

IFG = inferior frontal gyrus, DLPFC = dorsolateral prefrontal cortex, OFC = orbital frontal cortex.

a Pre-registered, confirmatory analyses.

In line with the pre-registered analysis plan, exploratory repeated measures analyses were conducted on the remaining channels in the fNIRS probe, to examine whether there were any additional channels showing significant block-type effects or interactions at 16 months (Route 4 of the analysis flow chart inFig. 3). Of the 32 channels included in the exploratory analyses, a total of 21 channels demonstrated a significant main effect of time in the HbO_2_and/or the HHb signal (reported inSupplementary Materials 5). Following correction for the FDR (64 comparisons; 32 channels × two chromophores), the main effect of time remained significant in 14 channels. Of the channels showing a significant main effect of time, a significant block-type effect was observed in the HHb signal in four channels covering the left superior parietal gyrus, the right inferior frontal gyrus (IFG), the left DLPFC, and the left OFC: (seeTable 4). These effects did not survive correction for the FDR (64 comparisons). SeeFigure 5below for*t*-statistic images of significant haemoglobin concentration differences between block types.

**Fig. 5.**
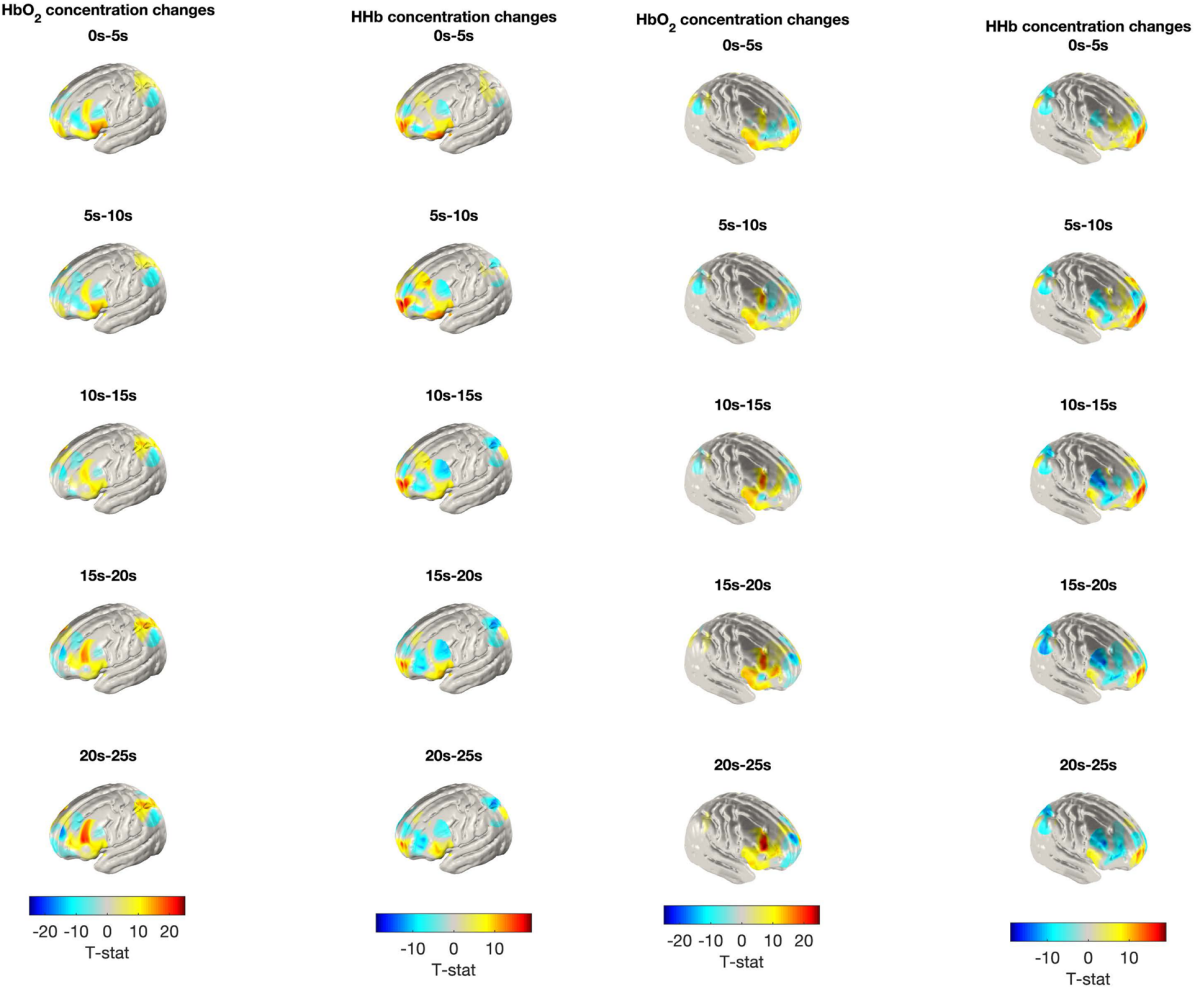
Group-level*T*-statistic image of the contrast in haemoglobin concentration changes between block types. Note. Group-level*T*-statistic images of the contrast in concentration changes of each chromophore between experimental and control blocks (experimental–control) in the left and right hemispheres. Images are displayed in the space of a cortical surface derived from averaged structural MRI data of a 12-month-old cohort of infants (Shi et al., 2011). Using this approach, all displayed*T*-statistic values are significantly different across block types (paired*t*-test) at the alpha level of*p*< .01. A positive*t*-stat value indicates that the mean chromophore concentration during a particular 5 s window is significantly higher than at baseline, though it does not necessarily require that these changes are associated with a canonical haemodynamic response. A negative*t*-stat value indicates that the mean chromophore concentration is significantly lower than at baseline. A positive*t*-stat value for changes in HbO_2_concentration is a marker of functional activation, while a negative*t*-stat value for changes in HHb is a marker of functional activation.

#### 3.2.1 Time course of the block-type effects

The purpose of these analyses was to examine the time course of the significant block-type effect (change over time bins). We had no prior expectations about the time course of the effect. To examine the time course of the significant block-type effects, paired*t*-tests were conducted on the six channels identified in the primary analyses (confirmatory and exploratory) as showing significant block-type effects. Results are reported inSupplementary Materials 6and visualised below inFigure 5. Following examination of the HRF patterns for each channel (seeFig. 6), it was decided that for the channel overlying the right DLPFC (Channel 26), any further analyses would be considered preliminary, and interpretations made very cautiously. This is because the only significant effects in this channel were observed in the first 5 s of the block time course and were not observed again afterwards. Additionally, the HRF in the first 5 s of the block (where the significant effect was observed) was inverted, such that there was an increase in deoxygenated haemoglobin and a decrease in oxygenated haemoglobin.

**Fig. 6.**
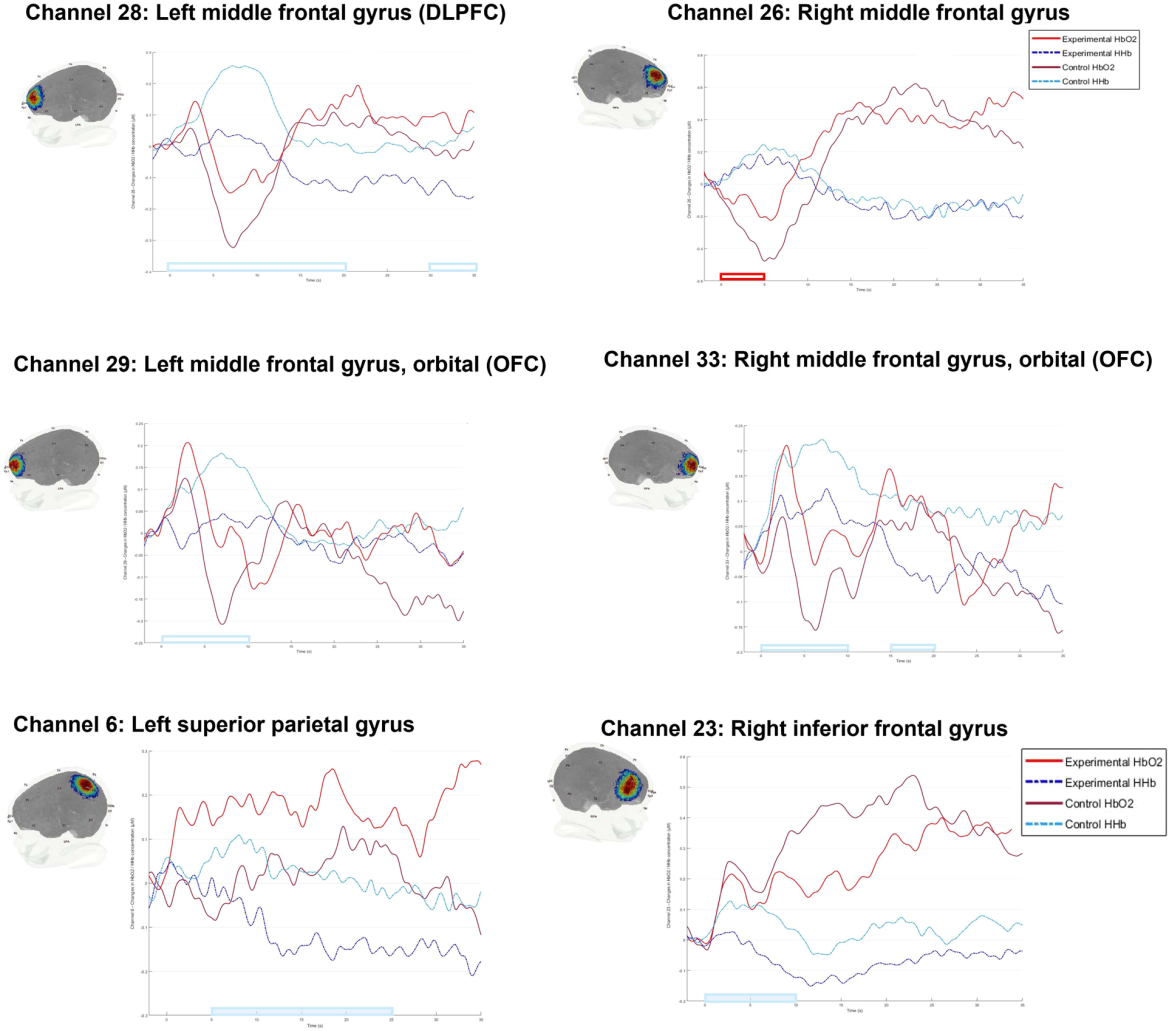
Haemodynamic response function for channels showing significant block-type effects.*Note.*This figure illustrates the average haemodynamic response function from -2 s (baseline) to 35 s of the block time course for channels showing significant block-type effects. The change in concentration in HbO_2_and HHb (μM) for each channel is represented on the y-axis, time bin is plotted on the x-axis. Bars (blue = HHb, red = HbO_2_) on the x-axis indicate the time bins where there is a significant block-type effect (note that these time-bin effects did not survive the correction for the FDR (7 comparisons)). Sensitivity profiles illustrate the position of each channel on the cortex and the channel sensitivity (heat map: red = more sensitive, blue = less sensitive)

#### 3.2.2 Description of the significant block-type effects

As seen inTable 4, a significant block-type effect was found in the HHb signal for six channels covering the left superior parietal gyrus, the right IFG, the bilateral DLPFC, and the bilateral OFC. The shape of the HRFs (Fig. 6) for channels covering the left superior parietal gyrus (Channel 6) and the right IFG (Channel 23) appears to follow the canonical pattern (a general increase in HbO_2_and decrease in HHb over time). Across the time course, there was a significantly greater decrease in HHb concentration in experimental blocks compared with in control blocks.

However, the HRF for channels overlying the bilateral DLPFC (Channels 26 and 28) and bilateral OFC (Channels 29 and 33) shows a different pattern. An inverted HRF can be seen in these channels at the beginning of the time course, such that there is a decrease in HbO_2_and an increase in HHb. Whilst this is different from the canonical HRF, this inverted response is often reported in infant research (Gemignani & Gervain, 2021) and may be related to the immature neurovascular system in the infant brain (Kozberg & Hillman, 2016). This inverted response can be clearly seen in the right DLPFC (Channel 26), however, as previously mentioned; since the only significant HbO_2_block-type effect was observed during this inversion in the first 5 s of the block time course, this effect should be considered with caution. The inverted HRF in the left DLPFC (Channel 28) occurs after an initial increase in HbO_2_and appears more pronounced than in the right DLPFC (Channel 26). It is during this inversion that there is a significant HHb block-type effect in the expected direction (lower HHb concentration in experimental blocks). However, this significant HHb block-type effect also continues after the inversion (15–20 s) and again at the end of the block time course (30–35 s) in the expected direction.

The HRF for channels overlying the bilateral OFC (Channels 29 and 33) also shows this inverted response. There is a significant HHb block-type effect from 0 to 10 s in the left OFC (Channel 29). In the first time bin (0–5 s), there is an increase in HbO_2_from baseline in both block types and an increase in HHb in the control block, but a decrease in HHb in experimental blocks. These early fluctuations may be a result of artifact or due a slower/immature neurovascular response in infants, as mentioned above. However, the HRF pattern during the significant HHb block-type effect in the next time window (5–10 s) appears to follow a more classic inverted response, where there is a decrease in HbO_2_and an increase in HHb. Despite the inversion, the block-type effect in this time window is in the expected direction as there is less HHb during experimental than control blocks. Similarly, in the right OFC (Channel 33), there is also a significant HHb block-type effect in the first 10 s. However, the significant effect in the first time bin (0–5 s) may be as a result of artifact or immature neurovascular coupling, as it can be seen that in the first 3–4 s, there is an increase in both the HbO_2_and HHb signal from baseline. The next significant time window (5–10 s) appears to show a classic inverted response whereby there is a decrease in HbO_2_and an increase in HHb. The HRF in the final time window showing a significant HHb effect (15–20 s) appears to be more canonical, with an increase in HbO_2_and a decrease in HHb (greater for experimental than for control blocks).

### 3.3 Individual differences analyses

In line with the pre-registration, confirmatory correlational analyses were conducted to investigate whether individual differences in neural activation in channels showing significant block-type effects across the identified time bins were associated with individual performance differences in the accuracy inhibitory score. Contrary to prediction, no significant associations were found (smallest*p*= .072; Channel 33, largest*p*= .840; Channel 23). Full results are reported inSupplementary Materials 6.

### 3.4 Non-preregistered exploratory fNIRS analyses

It was stated in the pre-registered analysis plan that we would only consider channels that showed a significant main effect of time when investigating block-type effects. However, upon inspecting the activation in individual channels, it was clear that some of the channels adjacent to newly identified channels showing a block-type effect (reported inSection 3.2) had a robust block-type effect (difference between experimental and control blocks) from very early on in the block time course (significant block-type effect but no effect of time). We felt that it was relevant to report these channels, especially given the qualitatively different shape of the channel activation profiles at 16 months compared with 10 months (addressed in Section 3.2.2.).

These channels covered the right parietal cortex and the right IFG; seeTable 5, and seeSupplementary Materials 7for HRF plots for these channels. Exploratory paired*t*-tests were conducted on these three channels to investigate the time course of the block-type effects; results are reported inSupplementary Materials 7. The activation found in these channels, whilst clustering in the same regions as other channels showing significant block-type effects, was based on non-planned exploratory analyses (not pre-registered) and the effects did not survive the procedure for controlling the FDR. As such, these results will be treated as preliminary but may be of interest in future replication work or other studies investigating the neural correlates of early inhibitory control.

**Table 5.** Preliminary (not pre-registered) HHb block-type effects.

Location	Hemisphere	Channel	Signal	Main effect of block type
Inferior parietal (angular gyrus)	Right	8	HHb	*F* (1, 35) = 5.000, *p* = .032, ηp ^2^ = .116
IFG	Right	20	HHb	*F* (1, 41) = 4.087, *p* = .050, ηp ^2^ = .090
	Right	31	HHb	*F* (1, 42) = 13.681, *p* <.001, ηp ^2^ = .246

*Note.*These are preliminary effects as channels did not show a significant main effect of time. These block-type effects did not survive the procedure for controlling the false discovery rate (64 comparisons). IFG = inferior frontal gyrus, DLPFC = dorsolateral prefrontal cortex.

### 3.5 Exploratory longitudinal brain changes from 10 to 16 months

It was stated in the pre-registered analysis plan that preliminary exploratory analyses would be conducted on the longitudinal fNIRS data to examine change in brain activation across assessment points. A total of 102 participants were included in the linear mixed models analyses (*N*= 59 from the 10-month assessment point,*N*= 43 from the 16-month assessment point), although note that only 18 participants contributed valid fNIRS data to both assessment points. As such, it is important to acknowledge that these analyses are limited in their statistical power due to the attrition at the 16-month assessment point resulting from the COVID-19 pandemic. Because of this, these results are reported in full inSupplementary Materials 8, however, a summary is provided below.

When examining the longitudinal change in the nine channels identified as showing significant block-type effects at 16 months, results of linear mixed models indicated that activation (HHb difference, i.e., greater HHb decrease in experimental compared with control blocks) in the right inferior parietal cortex (Channel 8), left DLPFC (Channel 28), and the right IFG orbital (Channel 31) increased significantly from 10 to 16 months (full results reported inSupplementary Materials 8). This suggests a greater recruitment of those regions when inhibition is required at 16 months compared with 10 months. When examining the longitudinal change in the six channels that showed significant block-type effects at 10 months, but not at 16 months, it was found that activation in the right DLPFC (Channel 25) decreased significantly from 10 to 16 months (seeSupplementary Materials 8for full results). This suggests that the right DLPFC is recruited less when inhibition is required at 16 months compared with 10 months. However, these preliminary exploratory results did not survive the correction for the FDR and should be considered with caution.

## 4 Discussion

### 4.1 Summary of key findings

The aim of the current study was to identify the neural correlates of response inhibition across the transition from late infancy to early toddlerhood. In brief, this study found preliminary evidence that 16-month-old toddlers activate regions of the bilateral PFC and parietal cortex when inhibition is required. This study used the same experimental paradigm and analysis methods as our previous study with the same participant sample at 10 months of age (Fiske et al., 2022). The behavioural results replicated our previous findings in that participants were significantly more accurate and made significantly faster (correct) responses on prepotent trials than on inhibitory trials (Fiske et al., 2022;Hendry et al., 2021;Holmboe et al., 2021). Furthermore, the longitudinal study design enabled us to investigate developmental change in the neural correlates of response inhibition across the transition from infancy to toddlerhood. Our results suggest that whilst response inhibition performance on the ECITT does not change from 10 to 16 months of age, the functional neural substrates that support response inhibition become more widespread (i.e., recruitment of the left hemisphere and of more inferior regions of the right prefrontal cortex) across the transition to the second year of life. We interpret these results cautiously because we applied stringent correction for multiple comparisons using the FDR (correcting for up to 64 tests given the number of channels in our fNIRS probe), and most results did not survive this correction. Nonetheless, since we had a reasonable sample size for a toddler fNIRS study, and research investigating combined neural and behavioural changes in response inhibition across this important developmental transition is currently very limited, we believe that these results will be of interest to the field. Pending replication and further work with this age group, we tentatively propose that the functional neural mechanisms mature*in advance of*improvement in response inhibition performance, which may occur later in the second year.

### 4.2 The neural correlates of response inhibition in the transition to the toddlerhood

Results of the pre-registered analyses suggested that channels overlying the left superior parietal gyrus, the right IFG, the bilateral DLPFC and the bilateral OFC were more active in blocks where inhibition was required than when no inhibition was required. Interestingly, two channels overlying the right DLPFC and right OFC that were found to show significant block-type effects at 10 months also showed these effects at 16 months, suggesting consistency in the recruitment of these regions during inhibitory-demanding blocks of the ECITT across this age period. However, the other four channels identified as showing a block-type effect at 10 months did not show this effect at 16 months. Unlike at 10 months, no significant brain-behaviour associations were found between the accuracy inhibitory score on the ECITT and neural activation in channels showing block-type effects at 16 months. Questions surrounding brain-behaviour associations warrant further investigation in studies with larger samples.

As evidenced by the longitudinal results of the current study, the activation of prefrontal regions in the right hemisphere appears to expand from the DLPFC to also include the IFG by 16 months of age. Results indicated that channels covering the right IFG (Channel 23, Channel 20, Channel 31) showed significantly greater activation when inhibition was required, and exploratory longitudinal analyses (reported inSupplementary Materials) revealed a significant increase in HHb activation in a channel overlying the right IFG orbital (from 10 to 16 months). This is consistent with substantial evidence in the literature that highlights the right IFG as a key neural substrate in response inhibition in adults (for review, seeAron et al. (2004,2014)) and that demonstrates the increasing importance of the right IFG with age (Rubia et al., 2007).

Similarly, we showed that the recruitment of prefrontal regions in the left hemisphere (alongside right frontal regions) appears to become increasingly important during response inhibition between 10 and 16 months. The involvement of the bilateral parietal and frontal cortices has been demonstrated in other fNIRS studies examining inhibitory control in older toddlers (Kerr-German et al., 2022), pre-schoolers (Li et al., 2017) and young children (Mehnert et al., 2013;Zhou et al., 2022). For example, in the study with participants closest in age to the current study,Kerr-German et al. (2022)found that stronger fNIRS functional connectivity between channels covering the left and right parietal cortex, and between channels covering the left PFC and left parietal cortex, was associated with better inhibitory control performance in 2.5-year-old toddlers. This evidence demonstrates the involvement of both hemispheres during an inhibitory control task in toddlerhood and suggests that functional connectivity between bilateral frontal and parietal regions may support the exertion of inhibitory control.

Overall, our results demonstrate that the emergence of response inhibition in the first year of life is supported by right-lateralised regions of frontal and parietal cortices (Fiske et al., 2022), but during the transition to toddlerhood, frontal and parietal regions in the left hemisphere are recruited alongside these right-lateralised regions when inhibition is required. Further, this study suggests that the recruitment of right frontal regions expands from 10 to 16 months to now include the right IFG, a key neural substrate of mature response inhibition (Aron et al., 2004,^2014^). It is possible that the right IFG starts “taking over” (at least partially) from the right DLPFC as a key neural substrate around the middle of the second year of life, although this proposal will need confirmation in future research with older toddlers. More broadly, future research should examine the developmental trajectory of response inhibition and the associated neural substrates later in toddlerhood and in early childhood to better understand whether the same neural mechanisms support the improvement of response inhibition during this fundamental developmental period.

### 4.3 Limitations and future directions

A major limitation of the current study is the significant level of participant attrition that occurred due to multiple necessary lab closures during the COVID-19 pandemic. As such, many participants who had been recruited as part of the study (and participated in sessions at 10 months) were unable to return for test sessions at 16 months. This resulted in the sample size at 16 months being much smaller than originally intended, and as a consequence, the statistical power of this study to identify small or medium effects was limited. Additionally, there was a substantial amount of data attrition (~50%) in the fNIRS data, which further limited the sample size and statistical power. This was particularly true for the individual differences analyses, which could not robustly assess brain-behaviour associations because of insufficient statistical power. Typically, individual differences analyses need large samples of over 100 participants (Dubois & Adolphs, 2016) to reliably investigate brain-behaviour associations (de Haas, 2018). As such, it was not possible to detect brain-behaviour associations in the current sample (*N = *43), particularly as these associations (if present) are likely to be of a small effect size.

Following on from this, it must be acknowledged that many of the significant effects reported in this study did not survive the procedure for controlling the FDR (Benjamini & Hochberg, 1995). As well as the limited sample size and statistical power, the large number of comparisons in this study (mostly linked to the fNIRS analyses and relating to the number of channels in the fNIRS probe) meant that the results did not remain significant after these strict controls. As such, the results of this study must be considered preliminary and interpreted with caution until they have been replicated in larger samples. Research is already in progress within our team that aims to replicate the results of the current study with 16-month-old infants (and our previous work with 10-month-old infants;Fiske et al., 2022) in a larger longitudinal sample.

To address the attrition of fNIRS data in future research, attempts should be made to reduce session-specific attrition (e.g., poor fNIRS cap placement). Indeed, according to a recent meta-analysis (Baek et al., 2023), variables such as infant age, study design, hardware design, and signal quality can impact the average attrition rate in infant fNIRS studies. One of the largest problems in the current study was data loss due to poor cap placement, which is a factor that can be improved with practice and training. The second reason for attrition was the strict inclusion criteria and data quality criteria that were imposed on the fNIRS dataset. This was done for the reason of improving the signal-to-noise ratio (which is already poor due to the nature of infant brain responses and motion artifacts) but could be re-evaluated in future such that a better balance is achieved between signal quality and data attrition. Although a common issue in infant neuroimaging research, it should be acknowledged that the level of fNIRS attrition in the current study may have biased our sample in some unknown way. Since our behavioural results indicated that toddlers who did not have valid fNIRS data performed very similarly to toddlers who did have valid fNIRS data, it could be argued that our fNIRS sample was not too biased. However, the effect of attrition on sample characteristics should continue to be rigorously evaluated.

A potential further limitation of this work is the use of a 12-month head model (Shi et al., 2011) to localise channel positions at 16 months. Whilst an alternative approach would have been to use a more closely age-matched head model to ensure a potentially more accurate localisation process, we had difficulty finding a suitable alternative model and, therefore, decided to use the 12-month model that we had used previously; this also retained consistency when comparing the fNIRS data from the 10- and 16-month assessment points. Ideally, future research would use subject-specific structural MRI scans from the cohort of participants assessed with fNIRS to specify the head model, although this is costly and not always appropriate or feasible in infant research. Further, use of precise optode position recording devices such as the Polhemus digitizer, photogrammetry, and video-based methods (e.g.,Erel et al., 2021;Xia et al., 2023) is becoming increasingly feasible for use with infant participants and produce more accurate optode registrations.

The results of the current study and of previous work (Fiske et al., 2022) demonstrated considerable differences in the shape of the HRF observed at 10 and 16 months of age, including the presence of inverted responses at 16 months (i.e., decrease in HbO_2_and increase in HHb in response to task blocks). Since the neurovascular system undergoes significant development in infancy and early childhood, it is likely that neurovascular coupling does not function in the same way as in adult brains (Kozberg & Hillman, 2016;Siddiqui et al., 2017). Although the shape and time course of the haemodynamic response in infancy is less well understood (de Roever et al., 2018), it is widely reported that there is significant variability in the infant haemodynamic response, and there is evidence to suggest that this variability can be influenced by the age of infants, as well as experimental factors (Issard & Gervain, 2018). Since very little is understood about the development of the HRF and the physiological neurovascular coupling mechanisms in infancy and toddlerhood, more fNIRS research is needed in this field before we can fully understand the variable responses (i.e., HRF shape) to the same task design observed in this study.

It is possible that regions of the cortex (i.e., motor regions) that were not measured with the fNIRS probe used in the current study played a role in task performance. Furthermore, since it is unlikely that the brain regions identified in this study are working in isolation, an important next step for this research is to investigate functional connectivity between frontal and parietal regions. Indeed, previous studies have suggested that functional connections between frontal and parietal cortices strengthen with development to support the improvement of executive processes in toddlers and young children (Buss & Spencer, 2014,^2018^;Kerr-German et al., 2022;Mehnert et al., 2013). Functional connectivity analyses would allow us to elucidate whether the regions of the brain found to be active in the current set of studies are functionally connected, and also how functional connections between brain regions in the frontal and parietal cortices may change with age to support response inhibition development. Furthermore, such analyses would allow us to compare connectivity within and between functional networks at 10 and 16 months, in order to understand more about the potential reorganisation processes occurring during this period of early brain development, beyond activation profiles. However, since the ECITT was developed as a traditional block design task devised to isolate cortical areas involved in response inhibition, functional connectivity analyses were not performed in the current study. It is clear that examining functional connectivity between frontal and parietal regions during the ECITT is an important area for future research, the key to which will be the development of new methodological/statistical approaches for task-based functional connectivity analyses.

### 4.4 Strengths and Implications

In using the same methodological paradigm with the same sample of participants at both 10 and 16 months of age, the results of the current study add valuable knowledge to our limited existing understanding of developmental change in the neural substrate of inhibitory control over this key transitional period. Through this longitudinal dataset, it has been possible not just to examine the differences between infants’ performance and associated neural activation at 10 and 16 months, but also to really consider the neural*changes*from the first to the second year of life. The longitudinal findings of the current study demonstrate that although there is no behavioural change from 10 to 16 months of age (16-month olds make just as many inhibitory errors in the ECITT as do 10-month olds), there appears to be more widespread recruitment of brain regions within the PFC and parietal cortex when inhibition is required at 16 months (i.e., more bilateral recruitment, and involvement of more inferior areas of the right PFC). These results are exciting because they shed new light on the functional maturation of the PFC across the transition from infancy to toddlerhood, in the context of stable response inhibition performance, and will be of great interest to the field of developmental cognitive neuroscience.

## Supplementary Material

Supplementary Material

## Data Availability

Anonymised data associated with this manuscript will be made available on the project’s Open Science Framework (OSF) webpage upon publication. The custom MATLAB scripts used to analyse the fNIRS data will also be made available on the OSF page at the point of publication. The code for the original ECITT task and the blocked version of the ECITT used with fNIRS are available on Figshare [https://figshare.com/articles/software/ECITT_Web_App/13258814], and the stimuli can be downloaded from the OSF archive for the original ECITT task:https://osf.io/ytfdp/. SeeHolmboe et al. (2021)for details on how to access demo versions. The code used to analyse the fNIRS data (HomER2) is available at [https://www.nitrc.org/projects/homer2] and a MATLAB script of the pre-processing stream used in this study will be made available on the OSF page. The code used to produce the head model and reconstruct images has been developed and released via [http://www.github.com/DOT-HUB]. The reconstructed images presented in this paper will also be available as a MATLAB figure in the OSF project upon publication. We aim to have entirely open and transparent code, processing pipelines, protocols and data— please get in touch with A.F. or K.H. if there is something you cannot find in the resources listed above.
