# Supplementary material for: The neural correlates of response inhibition across the transition from infancy to toddlerhood: An fNIRS study
Source: Imaging Neurosci (Camb). 2024 Jun 28;2:imag-2-00206. doi: 10.1162/imag_a_00206 (PMC12272267; doi:10.1162/imag_a_00206)
Supplement: Supplementary Material [file imag_a_00206-supp.pdf]

The Neural Correlates of Response Inhibition across the Transition from Infancy to Toddlerhood: An fNIRS study

Supplementary Materials

Abigail Fiske*, Liam Collins-Jones, Carina de Klerk, Katie Y. K. Lui, Alexandra Hendry, Isobel Greenhalgh, Anna Hall, Henrik Dvergsdal, Gaia Scerif, Karla Holmboe

*Corresponding author: Abigail Fiske, Department of Experimental Psychology, University of Oxford, United Kingdom. Email: abigail.fiske@psy.ox.ac.uk

Note. The following supplementary materials correspond to the article (as titled above). See also our project on the Open Science Framework [https://osf.io/j3s7g/] for materials relating to the article, these supplementary materials, or the data to which these reports are associated. The materials in this project are under a CC-By Attribution 4.0 International license. Please cite the article if using any of these materials.

Contents

1. **Participant Demographics** 2
   1.1. **Pilot and Longitudinal Samples** 2
2. **fNIRS: Pre-processing, Channel Locations, Analyses, Head Modelling and Image Reconstruction** 4
   2.1. **Channel Map** 4
   2.2. **fNIRS Pre-processing in HomER2** 5
   2.3. **Head Modelling, Channel Localisation and Image Reconstruction** 5
   2.4. **Anatomical Labels of Channels** 7
   2.5. **Overview of the fNIRS Preprocessing and Analysis Pipeline** 9
3. **Description of Variables** 12
4. **Parametric Test Assumptions** 15
5. **Behavioural Results** 16
6. **fNIRS Group-Level Results: Main Effect of Time** 20
7. **Non-preregistered Exploratory fNIRS Analyses** 24
8. **Exploratory Longitudinal Brain Associations from 10- to 16-months** 29
   8.1. **Channels Showing Significant Block Type Effects at 16-months** 29
   8.2. **Channels Showing Significant Block Type Effects at 10-months** 33
9. **References** 35

1. Participant Demographics

A total of 103 participants (*N* = 55 male infants) attended their first in-person test session at 16 months, and 98 participants (*N* = 52 male infants) returned for the second test session approximately one week later. Demographic information for this sample is reported in **Supplementary Supplementary Table *1*** below.

Supplementary Table 1. Demographic information for the 16-month participant sample.

	N	Mean	SD	Min	Max
Infant's age at Session 1 (days)	103	488.86	7.05	475	514
Infant's age at Session 2 (days)	98	498.44	7.40	478	518
Mother's age (years)	91	33.68	4.40	19	47
Father's age (years)	90	35.44	5.17	26	53
Maternal education (years)	90	17.80	2.57	12	25
Paternal education (years)	88	17.18	3.49	11	30
**Infant Ethnicity**	*N*	%			
Mixed – Other	1	0.97			
Other White	17	16.51			
Prefer not to say	1	0.97			
White and Asian	1	0.97			
White and Black African	1	0.97			
White and Black Caribbean	1	0.97			
White and Chinese	1	0.97			
White and Mexican	1	0.97			
White British	68	66.02			
Information not provided	11	10.68			
**Total**	**92**				

Note. Parental age and education (years) was recorded at the 10-month assessment point of the Oxford Early Executive Functions study.

1.1. Pilot and Longitudinal Samples

As stated in the main manuscript, the participant sample at 16-months was limited in that data collection had to stop prematurely because of the COVID-19 pandemic. As such, some participants who were tested at 10-months were unable to return for their session at 16-months (N ~ 66), and so the sample size was smaller than originally planned at this assessment point. To maximise statistical power at this time point, 16-month data from an additional 25 participants are included in the current sample. These participants were recruited as part of pilot test sessions for the longitudinal study and were assessed under the same administration protocol as participants in the longitudinal study at 16-months. Many of these pilot participants contributed behavioural ECITT data when they were 10-months old (reported in Hendry et al., 2021), however did not contribute blocked ECITT and / or fNIRS data to our previous publication (Fiske et al., 2022) because the blocked ECITT task was completed under a different administration protocol at this point in the piloting process (i.e., when the pilot participants were 10 months old). At 16-months, the ECITT protocol for both versions of the task was identical across the two samples. Please see **Supplementary Figure 1** below for a visual overview of the different samples and administration protocols.

Supplementary Figure 1. Overview of Samples in the Current Study.

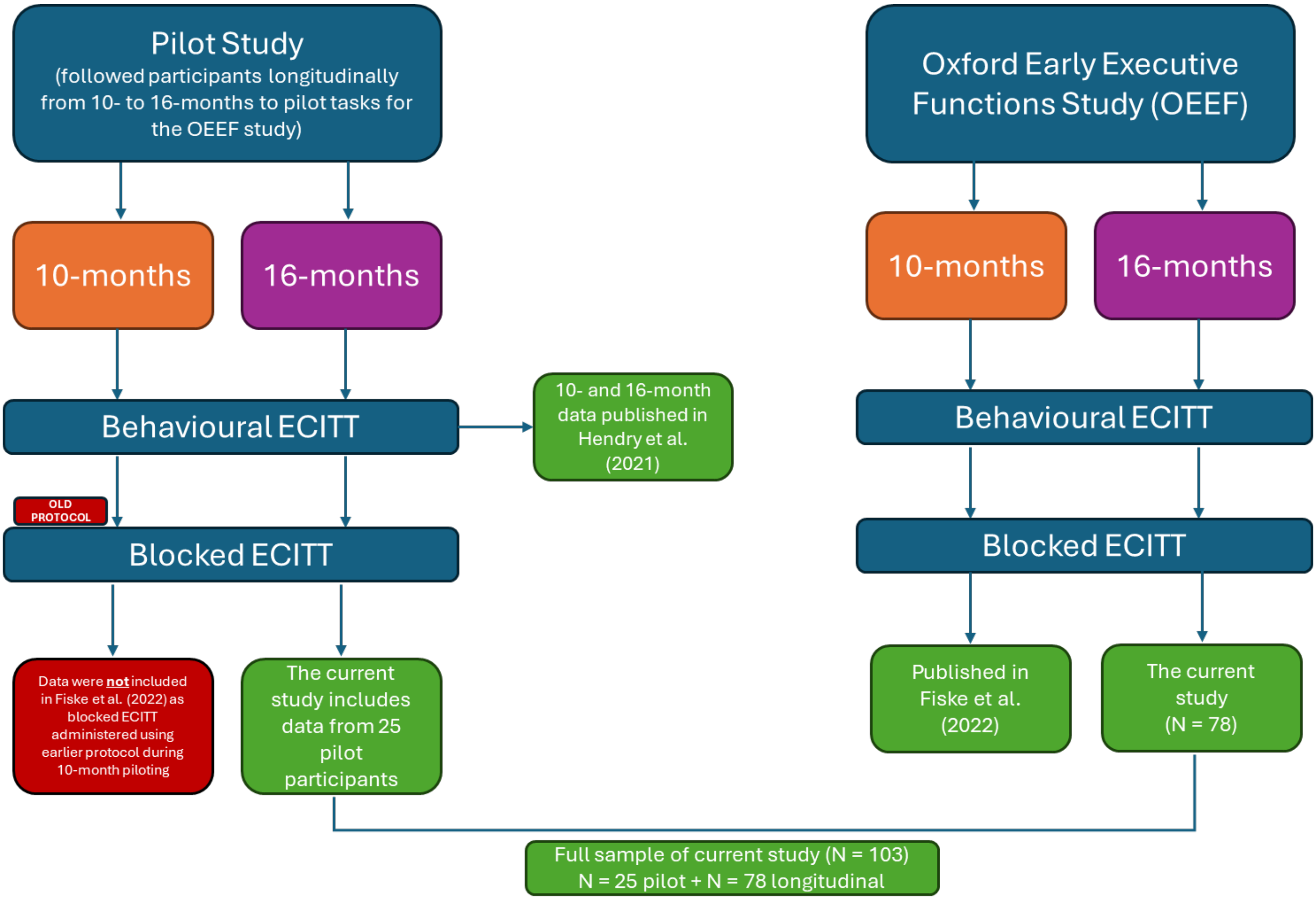

2. fNIRS: Pre-processing, Channel Locations, Analyses, Head Modelling and Image Reconstruction

2.1. Channel Map

A channel map is provided in **Supplementary Figure *2*** below that displays the position and channel numbers of the fNIRS probe associated with this research. Note that this is identical to that used at 10-months (as reported in Fiske et al., 2022). The black lines (representing channels) were manually added to this figure for visualisation purposes, and so are not scaled accurately. See **Section 2.3** below for the anatomical labels of the channels in the fNIRS probe.

Supplementary Figure 2. Channel Map of the fNIRS probe.

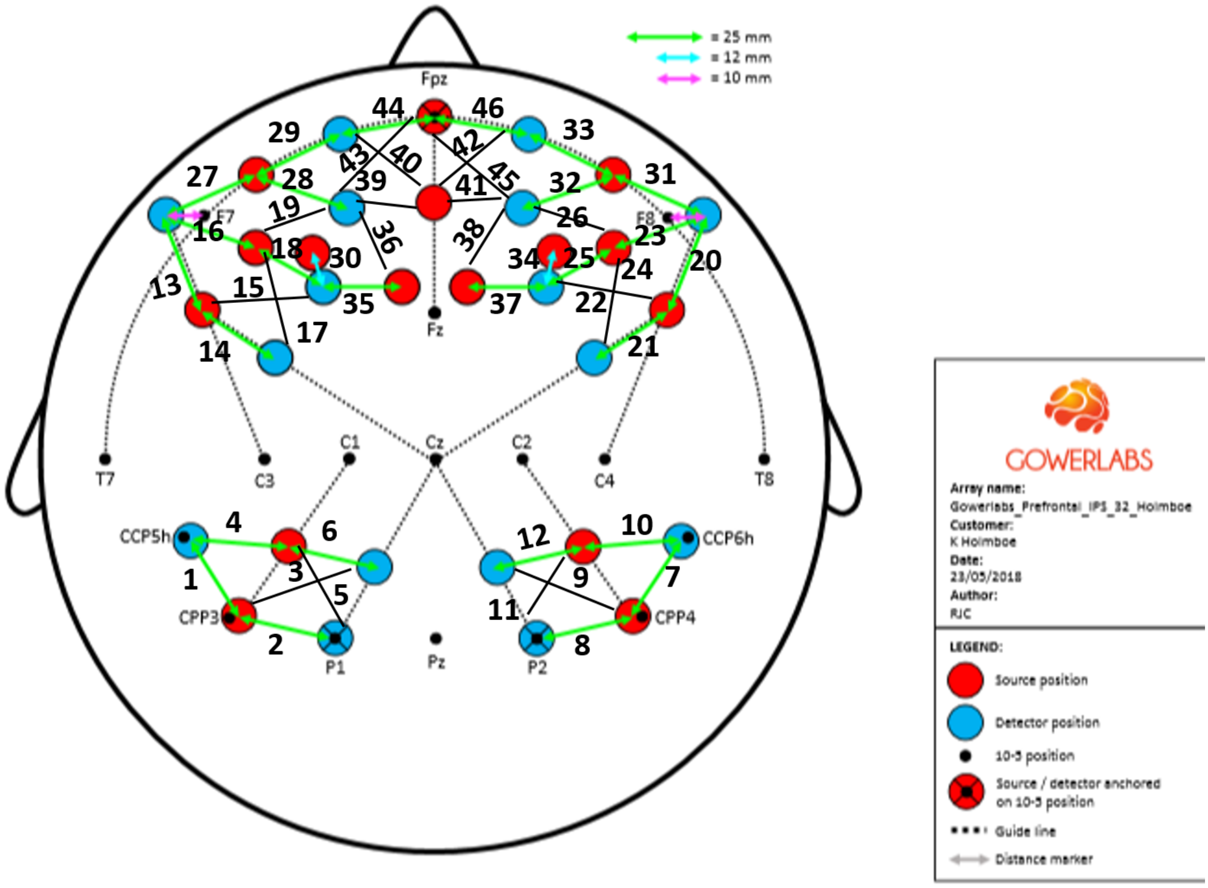

Note. Channels 1 – 12 overlay regions of the bilateral intraparietal sulcus (parietal cortex) and Channels 13 – 46 overlay regions of the bilateral prefrontal cortex. The source-detector separation between each nearest neighbour pair is 25mm, which is sufficient to sample cortical activity in infants (Lloyd-Fox et al., 2010; Taga et al., 2007).

2.2. fNIRS Pre-processing in HomER2

Data were pre-processed in HomER2 (Huppert et al., 2009). The processing functions and parameters we used in this study are reported below in **Supplementary Supplementary Table *2***. A .cfg file of this processing stream is available on OSF (https://osf.io/t9z6f).

Supplementary Table 2. HomER2 processing stream parameters.

hmrIntensity2OD		
enPruneChannels	dRange	1e-03 1e+03
	SNRthresh	2
	SDrange	0.0 45.0
	reset	0
hmrMotionArtifactByChannel	tMotion	1.0
	tMask	1.0
	STDEVthresh	15.5
	AMPthresh	0.40
hmrMotionCorrectSpline	p	0.99
	turnon	1
hmrMotionCorrectWavelet	iqr	1.5
	turn_on	1
hmrMotionArtifactByChannel	tMotion	1.0
	tMask	1.0
	STDEVthresh	15.5
	AMPthresh	0.40
hmrBandpassFilt	hpf	0.010
	lpf	0.80
hmrOD2Conc	ppf	5.2 4.8
enStimIncData_varagin	1	0.0 0.0
hmrBlockAvg	trange	-2.0 35.0

2.3. Head Modelling, Channel Localisation and Image Reconstruction

The below section of text has been taken from Section 2.6.5. of our previous publication (full reference below). Note that the text in italics is from the Supplementary Materials of this previous publication and is pasted here for completeness.

Fiske, A., de Klerk, C., Lui, K. Y., Collins-Jones, L., Hendry, A., Greenhalgh, I., ... & Holmboe, K. (2022). The neural correlates of inhibitory control in 10-month-old infants: A functional near-infrared spectroscopy study. *NeuroImage, 257,* 119241. https://doi.org/10.1016/j.neuroimage.2022.119241

To allow us to visualise the data on an age-appropriate head template, a model of the infant head was produced from averaged structural MRI data of a 12-month-old cohort (Shi et al., 2011). *Group-level tissue masks were combined to produce a mask of the spatial distribution of the cerebral tissues (white matter, grey matter, and cerebrospinal fluid). The inner skull border was delineated by the outside boundary of the cerebral tissue mask, while the scalp surface was defined using the Betsurf procedure* (Jenkinson et al., 2005) *where the group-level T1-weighted MRI template was used as an input. All voxels situated between the inner skull border and the scalp surface were assigned to be extra-cerebral tissue; this is a combined label for scalp and skull, and is commonly done in infant head models due to the difficulty in discerning these two tissues in infant MRI data* (Brigadoi et al., 2019; Collins-Jones et al., 2021; Frijia et al., 2021)*. The resulting four-layer tissue mask (consisting of white matter, grey matter, cerebrospinal fluid and extra-cerebral tissue) was converted to a tetrahedral volume mesh and a grey matter surface mesh using the iso2mesh package;* Fang & Boas, 2009*, see iso2mesh.sourceforge.net).*

As demonstrated by Collins-Jones et al. (2021), assuming a constant head size and array position is a valid approach for an image reconstruction approach using infant fNIRS data. The head model was scaled to the group mean head circumference measurement of the 59 infants in this study with useable fNIRS data. The positions of sources and detectors were registered virtually to the scalp surface of the head model using the Homer2 spring relaxation mechanism (Aasted et al., 2015). To model the transport of near-infrared light through the head model, TOAST++ ((Schweiger & Arridge, 2014), see http://toastplusplus.org) was employed to produce a forward model for each wavelength. Using the group-level block-averaged optical density data, the forward model was then used to reconstruct a time-series of images of HbO2 and HHb concentration changes for each condition. Image reconstruction was constrained to the grey matter nodes of the volume mesh, as per previous topographic approaches (Boas et al., 2004; Boas & Dale, 2005). The resulting reconstructed images were mapped to the grey matter surface mesh. Data preparation, meshing, forward modelling, and reconstruction were facilitated by the DOT-HUB Toolbox (www.github.com/DOT-HUB).

The cortical positions for channels showing significant experimental effects (reported in Section 3.2 *of Fiske et al., 2022*) were determined using the forward model. For each channel, the sensitivity values from the forward model mapped to the grey matter surface were used to compute a weighted average of grey matter node positions; the nearest grey matter node to the weighted average position was determined. Using the infant automated anatomical labelling (AAL) atlas presented by Shi et al. (2011), the anatomical label of the nearest grey matter node was determined and was assigned as the cortical label of the channel.

From the pre-registration associated with the current paper: (https://osf.io/hpb4s):

Note: A small change to the head modelling and channel localisation procedure was made for the 16-month dataset, such that there are some minor differences between the procedure used to localise channels at 10- and 16-months; see section ‘Prior knowledge’ for full details. This means that the label for Channel 32 (which is positioned on the border between the ‘middle frontal gyrus’ and the

'middle frontal gyrus, orbital' regions, according to the infant automated anatomical labelling atlas; Shi et al., 2011) has changed from the 'middle frontal gyrus, orbital' (as used in the 10-month paper; Fiske et al., 2022) to the 'middle frontal gyrus' label. Nevertheless, the position of this channel is closer to the right orbital region than the previously identified channels of interest in the right dorsolateral region (Channels 25 and 26; Fiske et al., 2022). Therefore, for the purposes of the analyses planned in this pre-registration, we consider Channel 32 to be part of the OFC and will use this label throughout our pre-registration. This also retains consistency in labelling between the current pre-registration and Fiske et al. (2022). MATLAB figures of the channel positions on the cortex at both 10- and 16-months are available to view here.

2.4. Anatomical Labels of Channels

This work was completed by Dr Liam Collins-Jones for the pre-registered study associated with these Supplementary Materials. **Supplementary Supplementary Table *3*** below provides the anatomical label for each channel in the fNIRS probe used in the current study. Aside from Channel 32 (see above), the only other changes in anatomical labels from 10-months (Fiske et al., 2022) to 16-months were in Channels 17 and 21, which were labelled as covering the inferior frontal gyrus, triangular at 10-months, but the middle frontal gyrus at 16-months.

Supplementary Table 3. Anatomical labels of channels in the fNIRS probe at 16-months.

Channel	Hemisphere	Cortical Region
**1**	Left	'Inferior parietal gyrus (P2)'
**2**	Left	'Inferior parietal gyrus (P2)'
**3**	Left	'Superior parietal gyrus (P1)'
**4**	Left	'Inferior parietal gyrus (P2)'
**5**	Left	'Superior parietal gyrus (P1)'
**6**	Left	'Superior parietal gyrus (P1)'
**7**	Right	'Inferior parietal gyrus (P2)'
**8**	Right	'Angular gyrus (AG)'
**9**	Right	'Angular gyrus (AG)'
**10**	Right	'Inferior parietal gyrus (P2)'
**11**	Right	'Superior parietal gyrus (P1)'
**12**	Right	'Superior parietal gyrus (P1)'
**13**	Left	'Inferior frontal gyrus, triangular (F3T)'
**14**	Left	'Inferior frontal gyrus, triangular (F3T)'
**15**	Left	'Middle frontal gyrus (F2)'
**16**	Left	'Inferior frontal gyrus, triangular (F3T)'
**17**	Left	'Middle frontal gyrus (F2)'
**18**	Left	'Middle frontal gyrus (F2)'
**19**	Left	'Middle frontal gyrus (F2)'

**20**	Right	'Inferior frontal gyrus, triangular (F3T)'
**21**	Right	'Middle frontal gyrus (F2)'
**22**	Right	'Middle frontal gyrus (F2)'
**23**	Right	Inferior frontal gyrus, triangular (F3T)'
**24**	Right	'Middle frontal gyrus (F2)'
**25**	Right	'Middle frontal gyrus (F2)'
**26**	Right	'Middle frontal gyrus (F2)'
**27**	Left	'Inferior frontal gyrus, orbital (F3O)'
**28**	Left	'Middle frontal gyrus (F2)'
**29**	Left	'Middle frontal gyrus, orbital (F2O)'
**30**	Left	'Middle frontal gyrus (F2)'
**31**	Right	'Inferior frontal gyrus, orbital (F3O)'
**32**	Right	'Middle frontal gyrus (F2)'
**33**	Right	'Middle frontal gyrus, orbital (F2O)'
**34**	Right	'Middle frontal gyrus (F2)'
**35**	Left	'Superior frontal gyrus, dorsolateral (F1)'
**36**	Left	'Superior frontal gyrus, dorsolateral (F1)'
**37**	Right	'Superior frontal gyrus, dorsolateral (F1)'
**38**	Right	'Superior frontal gyrus, dorsolateral (F1)'
**39**	Left	'Superior frontal gyrus, dorsolateral (F1)'
**40**	Left	'Superior frontal gyrus, medial (F1M)'
**41**	Right	'Superior frontal gyrus, dorsolateral (F1)'
**42**	Right	'Superior frontal gyrus, medial (F1M)'
**43**	Left	'Superior frontal gyrus, dorsolateral (F1)'
**44**	Left	'Superior frontal gyrus, medial orbital (F1MO)'
**45**	Right	'Superior frontal gyrus, dorsolateral (F1)'
**46**	Right	'Superior frontal gyrus, orbital (F1O)'

Note. The anatomical labels for each channel were created using the infant automated anatomical labelling (AAL) atlas presented by (Shi et al., 2011). Work conducted by Dr Liam Collins-Jones.

2.5. Overview of the fNIRS Preprocessing and Analysis Pipeline

The purpose of this section is to provide a full step-by-step outline of the fNIRS preprocessing and analysis pipeline used in the current study. All of the below steps are also outlined in the pre-registration (https://osf.io/hpb4s). A flow chart that summarises the key processes is provided in **Supplementary Figure 3** for visualisation purposes.

1) Preprocessing in HomER2

First, the channel-level fNIRS data for each participant were pre-processed in HomER2. The full processing pipeline is provided in **Supplementary Table *2*** above, but is summarised below:

- Raw intensity data were converted to optical density data
- Channels with an optical density that was too high or too low (1e – 03, 1e + 03) were excluded from further processing and analyses
- Motion artifacts were identified at the channel level and corrected using Spline and Wavelet
- A band pass filter was applied at the channel level (high pass filter: 0.010, low pass filter: 0.80) to filter out physiological noise or other artifacts
- Optical density data were converted to haemoglobin concentration data
- Manually excluded events were removed from the data
- Data for each block-type (control, experimental) were averaged across a period of 37 seconds, which contained 2s of the preceding baseline and 35 seconds of the block time course.

2) Primary Statistical Analyses – Channel Level

Next, we conducted our primary statistical analyses on the pre-processed fNIRS data at the channel-level. As outlined in our pre-registration, we had several planned analyses routes depending on whether our pre-registered hypothesis was confirmed. Our hypothesis was that there would be a significant block-type effect in six channels covering the right parietal cortex (Channels 10 and 12), the right DLPFC (Channels 25 and 26) and the right OFC (Channels 32 and 33), such that activation was greater when inhibition was required in experimental blocks, compared to in control blocks where inhibition was not required. This hypothesis was based on the results of our previous study with 10-month-old infants (Fiske et al., 2022). The first step in our analysis was to conduct repeated measures ANOVAs on the six pre-registered channels specified above. The next step would depend on the results of the repeated measures ANOVAs. See **Figure 3** of the main manuscript for an illustration of our analysis approach.

- **Route 1: All six pre-registered channels showed a significant block-type effect**
    - Confirmatory analyses
    - We will conduct paired t-tests on all six channels to examine the time course of the block-type effect
    - We will conduct correlational analyses to examine the potential association between activation in all six channels and individual differences in task performance.

- **Route 2: Some of the pre-registered channels showed a significant block-type effect**
  - Confirmatory analyses
  - We will conduct paired t-tests on only the channels that showed significant block-type effects to examine the time course of the block-type effect
  - We will conduct correlational analyses to examine the potential association between activation in the channels showing significant block-type effects and individual differences in task performance.
- **Route 3: None of the six pre-registered channels showed a significant block-type effect**
  - No further analyses would be conducted on these six channels

Following this, we will then conduct exploratory analyses on the remaining channels in the probe that were not pre-registered.

- **Route 4: All remaining channels**
  - Exploratory analyses
  - We will conduct repeated measures ANOVAs to examine which of the remaining channels were showing significant block-type effects
  - We will conduct paired t-tests on all channels showing significant block-type effects to examine the time course of the block type effect
  - We will conduct correlational analyses to examine the potential association between activation in all significant channels and individual differences in task performance.

3) Head Modelling and Channel Localisation

In order to localise the channels in our fNIRS probe to anatomical regions on the head (and so provide an anatomical label for each channel number), we followed the procedure outlined in **Section 2.3.** of the current Supplementary Materials. This approach involved using a forward model and an age-relevant head model to provide anatomic labels for each channel position. A list of anatomical labels can be found in **Supplementary Table *3***.

4) Secondary Analyses for Image Reconstruction – Image Space

In order to visualise the significant block-type effects identified in our primary analyses, we conducted a secondary analysis to produce an image reconstruction of our results. This approach is described in detail in **Section 2.3.** of the current Supplementary Materials. In brief, for each participant the block-averaged changes in optical density data* are used (in combination with the forward model computed to determine channel location) to reconstruct a concentration change image (one for HbO_2, one for HHb) for each 5s time window during the block-averaged 35s time window. Then, the values at each node across participants during these 5s time windows are compared to a 2s baseline using a two-sample *t*-test to produce the image (**Figure 5** of the main manuscript).

[*] Note: Since these are linear processes, the block-averaged changes in optical density we have obtained would be exactly the same as though we had directly computed them without converting to and from concentration changes"

Supplementary Figure 3. Flow Chart of fNIRS Analytic Pipeline.

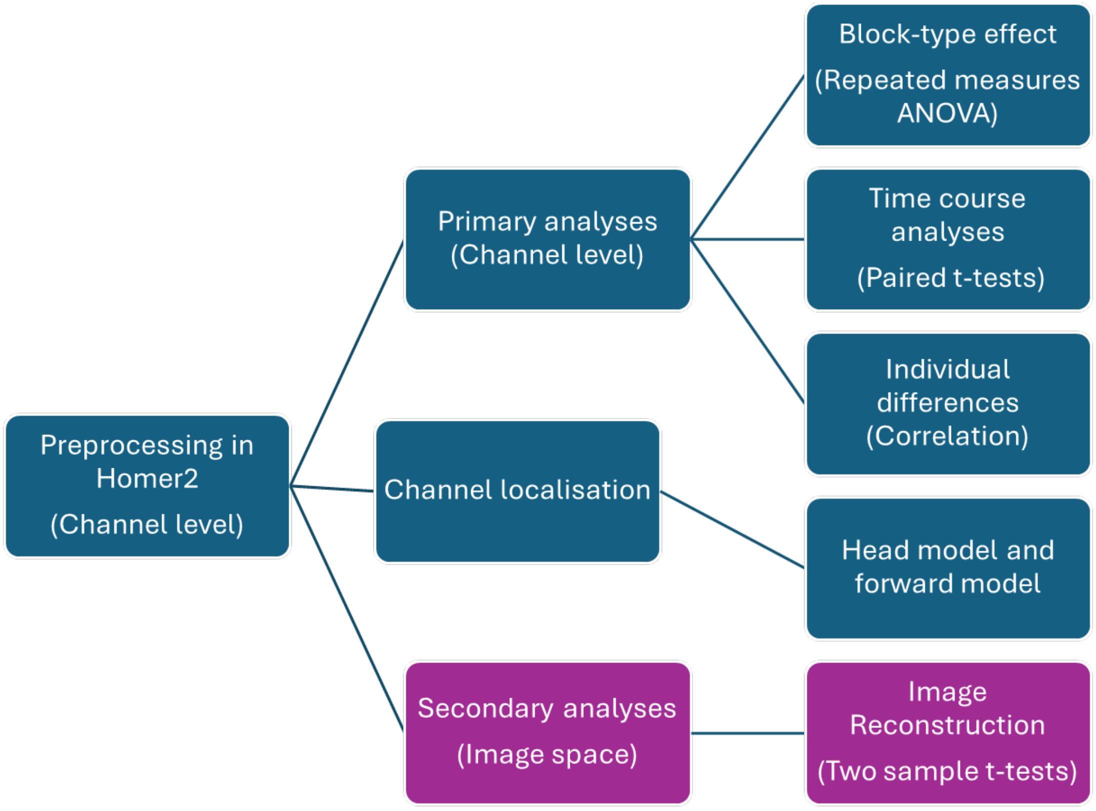

Note. This figure displays the analytic pipelines followed for the fNIRS data. Blue boxes denote channel-level analyses and purple boxes show analyses conducted in image space.

3. Description of Variables

The information provided below about the variables used in the current study has been taken from the pre-registration associated with this study (https://osf.io/hpb4s).

Measured variables

Behavioural data (from the ECITT):

- Prepotent trial accuracy: infants' accuracy on each valid* prepotent trial. Scored as 0 (incorrect) or 1 (correct).
- Inhibitory trial accuracy: infants' accuracy on each valid* inhibitory trial. Scored as 0 (incorrect) or 1 (correct).
- Prepotent trial reaction time (ms): calculated by the ECITT software from the onset of the trial until a touch is detected in the target response area (or as corrected during video coding* where the response was not detected by the iPad). Note that only valid and correct prepotent trials will be included in the reaction time analyses.
- Inhibitory trial reaction time (ms): calculated by the ECITT software from the onset of the trial until a touch is detected in the target response area (or as corrected during video coding* where the response was not detected by the iPad). Note that only valid and correct prepotent trials will be included in the reaction time analyses.

*See OSF document '16m_ECITT_CodingProtocol' for validity criteria and reaction time correction protocol.

fNIRS data:

The fNIRS hardware (Gowerlabs NTS system) measures a raw intensity signal (in two wavelengths: 780 and 850 nm) that then undergoes several transformations during pre-processing (e.g., conversion to optical density data and then to haemoglobin concentration data) to provide the measure of interest for our analysis: the average HbO_2 and HHb concentration per individual, per channel, per time bin.

- An average HbO_2 concentration variable will be generated for each participant with valid fNIRS data, for each time bin, for each channel (N ~46; although actual number may vary following exclusions).
- An average HHb concentration variable will be generated for each participant with valid fNIRS data, for each time bin, for each channel (N ~46; although actual number may vary following exclusions).

Behavioural indices (from the ECITT):

We will generate the same behavioural index of accuracy performance on the ECITT as described and used in our previous work (Fiske et al., 2022). This will be generated for both the behavioural and blocked versions of the ECITT.

- Mean prepotent accuracy: mean accuracy across all valid prepotent trials. Scored from 0 (all incorrect) to 1 (all correct).
- Mean inhibitory accuracy: mean accuracy across all valid inhibitory trials. Scored from 0 (all incorrect) to 1 (all correct).
- Accuracy inhibitory score*: The accuracy inhibitory score will be used as an index of infants' response inhibition performance such that a larger inhibitory score will be indicative of better response inhibition ability. In cases where mean prepotent accuracy is higher than mean inhibitory accuracy (mean prepotent accuracy > mean inhibitory accuracy), the accuracy inhibitory score will be calculated using the following formula: (1 – (mean prepotent accuracy – mean inhibitory accuracy)/mean prepotent accuracy).

  Based on previous results (Fiske et al., 2022; Hendry et al., 2021; Holmboe et al., 2021), most infants are expected to have higher accuracy on prepotent trials compared to inhibitory trials. In cases where mean inhibitory accuracy is higher than mean prepotent accuracy (mean inhibitory accuracy > mean prepotent accuracy), the accuracy inhibitory score will be calculated using a different formula: (1 – (mean prepotent accuracy – mean inhibitory accuracy) × mean prepotent accuracy). This alternative formula ensures that we avoid inflating the scores for infants who are more accurate on inhibitory than prepotent trials (i.e., are showing the opposite pattern than expected). Based on the data collected at 10-months* (Fiske et al., 2022), we expect that only a small number of infants in the 16-month dataset will have higher mean inhibitory accuracy than mean prepotent accuracy.

  *Note that in Fiske et al. (2022), the 'accuracy inhibitory score' was referred to as the 'inhibitory score'. Here, we did not use the alternative adjustment (multiplying by mean prepotent accuracy) for infants who had a higher mean inhibitory accuracy than mean prepotent accuracy (N = 13 of 121 participants, min difference = 0.01, max difference = 0.24). Instead, the same adjustment was used for all participants, as described above for the accuracy inhibitory score calculation, i.e.,: (1 – (mean prepotent accuracy – mean inhibitory accuracy)/mean prepotent accuracy). We have decided that for the current pre-registration of the 16-month dataset, for infants who have higher mean inhibitory accuracy than mean prepotent accuracy, we will apply the alternative adjustment (multiplying by mean prepotent accuracy instead of dividing by mean prepotent accuracy) to ensure that we are not unnecessarily inflating the scores of these participants who perform worse on the prepotent trials. We consider this a more accurate representation of individual accuracy performance, although it is likely to only make a minor difference to the results due to the small number of

participants showing the opposite effect and the small magnitude of this difference when it does occur.

We will additionally calculate:

- Median reaction time: This variable will be calculated for each trial type (prepotent and inhibitory) in each participant and will only include reaction times from correct and valid trials. The two indices will be labelled ‘prepotent median reaction time’ and ‘inhibitory median reaction time’. Individual reaction times shorter than 300 ms or longer than 5000 ms will be excluded before the median is calculated (as per Hendry et al., 2021).

Note that reaction time data was not used at 10-months (Fiske et al., 2022). For discussion of why we do not consider reaction time data collected from 10-month-old infants to be valid, see Lui et al. (2021) and Hendry et al. (2021).

4. Parametric Test Assumptions

All variables were tested for normality by examining the results of the Shapiro-Wilk test and the skewness (*z*-score) of the data; the results and conclusions of these tests are reported in **Supplementary Supplementary Table *4*** below. The significance value of the Shapiro-Wilk test informs whether the data are normally distributed; if $p > .005$, this suggests that the data are normally distributed, but if $p < .005$, this suggests that the data may deviate from a normal distribution. According to Kim (2013), for samples with more than 50 and less than 300 participants, a z-scored skew > 3.29 would suggest the sample distribution is not normal. This criterion has been applied to the behavioural variables used in this study when determining normality. In cases where variables are not normally distributed and are skewed beyond an acceptable level, non-parametric tests will be conducted alongside parametric tests to check for convergence. The fNIRS data were tested using Mauchly's test of sphericity and data from all but one channel (Channel 9) did not meet the sphericity assumption. Therefore, the Greenhouse-Geisser degrees of freedom and *p*-values were reported to account for the lack of sphericity in all channels.

Supplementary Table 4. Results of normality tests for the 16-month dataset

	Variable	*N*	Shapiro-Wilk (*p*)	Skew (z)	Conclusion
Session 1	Mean inhibitory accuracy	77	<.001	0.24	Not normally distributed, acceptable skew
	Mean prepotent accuracy	77	<.001	-6.06	**Not normally distributed, skew not acceptable**
	Accuracy inhibitory score	77	<.001	0.25	Not normally distributed, acceptable skew
	Median inhibitory reaction time	66	<.001	14.27	**Not normally distributed, skew not acceptable**
	Median prepotent reaction time	66	<.001	7.45	**Not normally distributed, skew not acceptable**
Session 2	Mean inhibitory accuracy	81	<.001	0.12	Not normally distributed, acceptable skew
	Mean prepotent accuracy	81	<.001	-5.84	**Not normally distributed, skew not acceptable**
	Accuracy inhibitory score	81	.018	0.82	Normally distributed, normal skew
	Median inhibitory reaction time	77	<.001	6.98	**Not normally distributed, skew not acceptable**
	Median prepotent reaction time	77	<.001	4.25	**Not normally distributed, skew not acceptable**
Individual Differences	HHb diff Channel 6 Bins 2 – 5	39	.123	1.78	Normally distributed, normal skew
	HHb diff Channel 23 Bins 1 – 2	43	.131	2.04	Normally distributed, normal skew
	HbO_2 diff Channel 26 Bin 1	37	.861	0.41	Normally distributed, normal skew
	HHb diff Channel 28 Bins 1 – 4 and Bin 7	42	.006	3.56	**Not normally distributed, skew not acceptable**
	HHb diff Channel 29 Bins 1 – 2	42	.023	2.17	Not normally distributed, acceptable skew
	HHb diff Channel 33 Bins 1, 2 & 4	43	.315	-.723	Normally distributed, normal skew

Note. Variables that are not normally distributed are highlighted in bold.

5. Behavioural Results

Since the mean prepotent accuracy and the median reaction time variables for both sessions were not normally distributed, non-parametric related-samples Wilcoxon signed rank tests were conducted. The results (**Supplementary Supplementary Table *5***) converged with the results of the parametric tests (reported in the manuscript).

Supplementary Table 5. Results of Wilcoxon signed-rank tests of trial type effects.

Accuracy	Test Statistic (*z*)	*P*	Effect size (*d*)
**Session 1**	6.186	**<.001**	0.79
**Session 2**	6.846	**<.001**	0.76
RT	**Test Statistic**	***P***	**Effect size (*d*)**
**Session 1**	-3.063	**.002**	0.42
**Session 2**	-4.697	**<.001**	0.54

Note. RT = reaction time. Statistically significant results are highlighted in bold.

<u>Behavioural Development from 10- to 16-months</u>

To examine behavioural development (mean accuracy) from 10- to 16-months, two separate repeated measures linear mixed models were carried out (one model for Session 1 data and one model for Session 2 data; 2 × 2: trial type, age). Results are reported in **Supplementary Supplementary Table *6*** below, and estimated marginal means are reported in **Supplementary Supplementary Table *7*** below.

Supplementary Table 6. Behavioural accuracy on the ECITT from 10- to 16-months.

Session 1	Test Statistic	*P*	Effect size (ηp^2)
**Trial type**	*F* (1, 64) = 168.030	**<.001**	.724
**Age**	*F* (1, 64) = .000	.983	.000
**Interaction**	*F* (1, 64) = 1.107	.294	.013
Session 2	**Test Statistic**	***P***	**Effect size (ηp^2)**
**Trial type**	*F* (1, 61) = 149.127	**<.001**	.710
**Age**	*F* (1, 61) = .109	.743	.002
**Interaction**	*F* (1, 61) = .093	.762	.002

Note. Statistically significant results are highlighted in bold.

Supplementary Table 7. Accuracy on the ECITT from 10- to 16-months; Estimated marginal means.

Session 1		Estimated marginal mean	Standard error	Confidence interval
**Trial type**	Inhibitory	.492	.031	.429, .555
	Prepotent	.912	.008	.897, .928
**Age**	10-months	.703	.021	.661, .744
	16-months	.702	.021	.660, .744
**Interaction**	Inhibitory 10-months	.508	.040	.429, .587
	Inhibitory 16-months	.476	.043	.390, .562
	Prepotent 10-months	.897	.012	.873, .922
	Prepotent 16-months	.928	.009	.910, .946
Session 2		**Estimated marginal mean**	**Standard error**	**Confidence interval**
**Trial type**	Inhibitory	.555	.026	.503, .608
	Prepotent	912	.009	.894, .930
**Age**	10-months	.729	.017	.695, .763
	16-months	.739	.022	.694, .783
**Interaction**	Inhibitory 10-months	.555	.035	.485, .625
	Inhibitory 16-months	.555	.043	.470, .641
	Prepotent 10-months	.903	.012	.880, .926
	Prepotent 16-months	.922	.011	.900, .944

Test re-test reliability and between session consistency

Pearson correlation analyses indicated significant test re-test reliability between mean inhibitory accuracy, accuracy inhibitory score and median prepotent reaction time in Session 1 and Session 2, as hypothesised (see **Supplementary Supplementary Table *8***). Contrary to expectations, the correlation coefficients for median inhibitory reaction time and mean prepotent accuracy were not significant. Since the median reaction time and mean prepotent accuracy variables were not normally distributed, Spearman's rho correlation tests were conducted to test for convergence. Results were consistent with the results of the Pearson's correlations: median inhibitory reaction time: r_s (54) = .165, p = .111, [CI = -.109, .394], median prepotent reaction time: r_s (54) = .287, p = .016, [CI = .009, .534], mean prepotent accuracy: r_s (65) = .090, p = .470, [CI = -.194, .339].

Contrary to hypotheses, results of the paired t-tests (**Supplementary Table 8**) indicated that the mean inhibitory accuracy and accuracy inhibitory score were significantly higher, and median reaction times were significantly faster in Session 2 than in Session 1. This aligns with the results of the repeated measures ANOVAs (reported in **Supplementary Table 9**, estimated marginal means reported in

Supplementary Table *10*). There was a significant main effect of session, indicating that infants were significantly more accurate, and responded significantly faster, on the blocked ECITT than on the behavioural ECITT. No significant trial type by session interaction effects were present.

Supplementary Table 8. ECITT test re-test reliability and between session consistency.

	Test re-test reliability			Between-session consistency		
	Correlation coefficient	*p*	Confidence interval	Test Statistic	*p*	Effect size (*d*)
**Mean inhibitory accuracy**	*r* (65) = .572	**<.001**	.403, .711	*t* (66) = -2.638	**.010**	-.322
**Mean prepotent accuracy**	*r* (65) = .082	.508	-.177, .368	*t* (66) = -1.193	.237	-.146
**Accuracy inhibitory score**	*r* (65) = .536	**<.001**	.375, .688	*t* (66) = -2.247	**.014**	-.274
**Median inhibitory RT**	*r* (54) = .216	.055	-.009, .430	*t* (55) = 2.832	**.003**	.378
**Median prepotent RT**	*r* (54) = .397	**.001**	.129, .583	*t* (55) = 3.645	**<.001**	.487

Note. RT = reaction time. Statistically significant results are highlighted in bold.
P-values for the accuracy inhibitory score and median RT variables are one-tailed (because these statistical tests were pre-registered).
P-values for the mean accuracy variables are two-tailed (because these statistical tests were not pre-registered).

Supplementary Table 9. ECITT between session consistency (repeated measures ANOVA).

Accuracy	**Test Statistic**	***P***	**Effect size (ηp^2)**
**Trial type**	*F* (1, 66) = 111.442	**<.001**	.628
**Session**	*F* (1, 66) = 9.712	**.003**	.128
**Interaction**	*F* (1, 66) = 3.841	.054	.055
RT	**Test Statistic**	***P***	**Effect size (ηp^2)**
**Trial type**	*F* (1, 55) = 28.483	**<.001**	.341
**Session**	*F* (1, 55) = 13.298	**<.001**	.195
**Interaction**	*F* (1, 55) = .795	.376	.014

Note. RT = reaction time. Statistically significant results are highlighted in bold.

Supplementary Table 10. ECITT between session consistency (estimated marginal means).

Accuracy		**Estimated marginal mean**	**Standard error**	**Confidence interval**
**Trial type**	Inhibitory	.525	.036	.454, .597
	Prepotent	.928	.007	.913, .942
**Session**	1	.698	.020	.657, .739
	2	.755	.019	.717, .793
**Interaction**	Inhibitory S1	.476	.043	.391, .561
	Inhibitory S2	.575	.038	.499, .651
	Prepotent S1	.920	.010	.900, .940
	Prepotent S2	.936	.009	.917 .954
RT		**Estimated marginal mean**	**Standard error**	**Confidence interval**

**Trial type**	Inhibitory	1549	47	1456, 1642
	Prepotent	1362	31	1300, 1423
**Session**	1	1547	49	1449, 1645
	2	1364	37	1290, 1438
**Interaction**	Inhibitory S1	1656	68	1520, 1791
	Inhibitory S2	1442	51	1340, 1544
	Prepotent S1	1438	44	1350, 1525
	Prepotent S2	1286	29	1228, 1344

Note. RT = reaction time (reported in milliseconds).

Sub-sample differences (Session 2)

To test whether performance significantly differed by trial type (inhibitory, prepotent) or between sub-samples (those with valid fNIRS data (N = 43) and those with only valid ECITT data (N = 38)), two separate 2 × 2 mixed ANOVAs were conducted on the accuracy and reaction time data from the blocked ECITT (Session 2). Estimated marginal means are reported in **Supplementary Supplementary Table *11***.

Supplementary Table 11. Estimated marginal means for mixed ANOVAs (Blocked ECITT).

Accuracy		**Estimated marginal mean**	**Standard error**	**Confidence interval**
**Trial type**	Inhibitory	.539	.036	.467, .610
	Prepotent	.924	.010	.904, .943
**Sub-sample**	fNIRS data	.711	.025	.662, .761
	No fNIRS data	.751	.027	.698, .804
**Interaction**	No data Inhibitory	.581	.052	.477, .686
	No data Prepotent	.921	.014	.893, .950
	Data Inhibitory	.496	.049	.398, .594
	Data Prepotent	.926	.013	.900, .953
RT		**Estimated marginal mean**	**Standard error**	**Confidence interval**
**Trial type**	Inhibitory	1459	47	1366, 1553
	Prepotent	1285	24	1238, 1332
**Sub-sample**	fNIRS data	1292	43	1297, 1378
	No fNIRS data	1452	46	1361, 1544
**Interaction**	No data Inhibitory	1549	68	1412, 1685
	No data Prepotent	1356	34	1287, 1424
	Data Inhibitory	1370	64	1242, 1498
	Data Prepotent	1214	32	1150, 1278

Note. RT = reaction time (reported in milliseconds). RT variables only include data from correct trials.

6. fNIRS Group-Level Results: Main Effect of Time

The results of the repeated measures ANOVA investigating which channels showed a significant change in haemoglobin concentration across the block time course (compared to baseline; main effect of time) are presented in **Supplementary Supplementary Table *12*** (HbO_2) and **Supplementary Supplementary Table *13*** (HHb).

Supplementary Table 12. Main effect of time (HbO_2).

	Left hemisphere			Right hemisphere		
	Channel	*F*	*p*	Channel	*F*	*p*
Intraparietal Sulcus	1	2.459	.084	7	4.981	**.007***
	2	2.681	.058	8	.518	.640
	4	3.693	**.019***			
	6	.368	.735			
Prefrontal Cortex	13	.517	.661	20	.766	.472
	14	4.897	**.007***	21	5.888	**.003***
	16	2.642	.068	23	3.367	**.035**
	17	13.292	**<.001***	24	24.795	**<.001***
	18	15.754	**<.001***	25	18.439	**<.001***
	19	13.232	**<.001***	26	18.025	**<.001***
	27	.188	.846	31	1.194	.311
	28	3.762	**.015***	32	6.252	**<.001***
	29	1.772	.168	33	.844	.460
	35	1.018	.376	37	1.730	.180
	36	2.130	.114	38	.308	.804
	39	7.696	**<.001***	41	2.642	.059
	40	6.958	**<.001***	42	4.981	**.005***
	43	6.529	**<.001***	45	2.813	**.048**
	44	9.078	**<.001***	46	7.153	**<.001***

Note. Statistically significant results are highlighted in bold. * = significant at $p < .05$, corrected for 84 comparisons (or 12 comparisons for confirmatory analyses (6 channels × 2 chromophores)). Note that 6 channels were pre-registered (Channels 10, 12, 25, 26, 32 and 33), but only four channels were included in analyses as less than 70% of participants contributed data to Channels 10 and 12.

Supplementary Table 13. Main effect of time (HHb).

	Left hemisphere			Right hemisphere		
	Channel	*F*	*p*	Channel	*F*	*p*
Intraparietal Sulcus	1	1.904	.146	7	1.754	.169
	2	.855	.427	8	1.614	.199
	4	3.715	**.024**			
	6	3.327	**.030**			
Prefrontal Cortex	13	1.260	.291	20	1.575	.201
	14	1.349	.261	21	4.685	**.006***
	16	1.484	.229	23	3.018	**.041**
	17	5.320	**<.001***	24	12.480	**<.001***
	18	8.959	**<.001***	25	15.672	**<.001***
	19	10.989	**<.001***	26	15.028	**<.001***
	27	.744	.504	31	1.328	.267

28	7.177	**<.001***	32	9.998	**<.001***
29	3.418	**.026**	33	5.075	**.003***
35	1.757	.166	37	1.872	.152
36	5.196	**.003***	38	1.861	.163
39	4.936	**.008***	41	8.469	**<.001***
40	5.128	**.006***	42	6.926	**<.001***
43	3.519	**.033**	45	4.453	**.005***
44	1.724	.175	46	1.139	.334

Note. Statistically significant results are highlighted in bold. * = significant at $p < .05$, corrected for 84 comparisons (or 12 comparisons for confirmatory analyses (6 channels × 2 chromophores)). Note that 6 channels were pre-registered (Channels 10, 12, 25, 26, 32 and 33), but only four channels were included in analyses as less than 70% of participants contributed data to Channels 10 and 12.

fNIRS Time Course Analyses

To examine the time course of the significant effects, paired t-tests were conducted on the six channels identified in the primary analyses as showing significant time and block-type effects; results are reported in **Supplementary Supplementary Table *14*** below.

Supplementary Table 14. Time course of the significant HbO_2 or HHb block type effects

Location	Channel	Signal	0 – 5s	5 – 10s	10 – 15s	15 – 20s	20 – 25s	25 – 30s	30 – 35s
**Left superior parietal gyrus**	6	HHb	t (38) = .713, p = .480, d = .114	**t (38) = 2.051, p = .047, d = .328**	**t (38) = 2.385, p = .022, d = .382**	**t (38) = 2.089, p = .043, d = .335**	**t (38) = 2.083, p = .044, d = .334**	t (38) = 2.013, p = .051, d = .322	t (38) = 1.808, p = .078, d = .290
**Right IFG**	23	HHb	**t (42) = 2.285, p = .027, d = .348**	**t (42) = 2.293, p = .027, d = .350**	t (42) = 1.364, p = .180, d = .208	t (42) = 1.777, p = .083, d = .271	t (42) = 1.634, p = .110, d = .249	t (42) = .974, p = .336, d = .148	t (42) = 2.011, p = .051, d = .307
**Left DLPFC**	28	HHb	**t (41) = 2.454, p = .018*, d = .379**	**t (41) = 3.192, p = .003*, d = .493**	**t (41) = 2.738, p = .009*, d = .423**	**t (41) = 2.069, p = .045, d = .319**	t (41) = 1.526, p = .135, d = .235	t (41) = 1.728, p = .092, d = .267	**t (41) = 2.567, p = .014*, d = .396**
**Right DLPFC**	26	HbO_2	**t (36) = -2.040, p = .049, d = -.335**	t (36) = -1.926, p = .062, d = -.317	t (36) = -1.039, p = .306, d = -.171	t (36) = -.312, p = .757, d = -.051	t (36) = .998, p = .325, d = .164	t (36) = .124, p = .902, d = .020	t (36) = -1.506, p = .141, d = -.248
**Left OFC**	29	HHb	**t (41) = 2.764, p = .009*, d = .427**	**t (41) = 2.676, p = .011*, d = .413**	t (41) = 1.482, p = .146, d = .229	t (41) = 1.013, p = .317, d = .156	t (41) = 1.134, p = .263, d = .175	t (41) = .835, p = .409, d = .129	t (41) = 1.504, p = .140, d = .232
**Right OFC**	33	HHb	**t (42) = 2.031, p = .049, d = .310**	**t (42) = 2.119, p = .040, d = .323**	t (42) = 1.314, p = .196, d = .200	**t (42) = 2.085, p = .043, d = .318**	t (42) = 1.546, p = .130, d = .236	t (42) = 1.236, p = .223, d = .189	t (42) = 1.945, p = .058, d = .297

Note. Statistically significant results are highlighted in bold. * = significant after correcting the FDR (7 comparisons). IFG = inferior frontal gyrus, DLPFC = dorsolateral prefrontal cortex, OFC = orbital frontal cortex.

Individual Differences Analyses

In line with the pre-registration, confirmatory correlational analyses were conducted to investigate whether individual differences in neural activation in channels showing significant block type effects across the identified time-bins were associated with individual performance differences in the accuracy inhibitory score. Contrary to prediction, no significant associations were found (**Supplementary Supplementary Table *15***).

Supplementary Table 15. Individual differences analyses.

Location	Hemisphere	Channel	Signal	Time Bins	Statistic
**Superior parietal gyrus**	Left	6	HHb	2 – 5	r (37) = -.234, p = .151, CI = [-.468, .033]
**IFG**	Right	23	HHb	1 – 2	r (41) = -.032, p = .840, CI = [-.289, .261]
**DLPFC**	Left	28	HHb	1, 4, 7	r (40) = -.212, p = .177, CI = [-.478, .140]
**OFC**	Left	29	HHb	1 – 2	r (40) = -.070, p = .661, CI = [-.356, .245]
	Right	33	HHb	1, 2, 4	r (41) = .227, p = .072, CI = [-.112, .518] [a]

Note. [a] = Confirmatory analysis. Where multiple time bins are included, averages of the haemoglobin difference variables have been calculated from the haemoglobin difference value within each individual time bin showing a significant effect of block type for that channel.
IFG = inferior frontal gyrus, DLPFC = dorsolateral prefrontal cortex, OFC = orbital frontal cortex.

7. Non-preregistered Exploratory fNIRS Analyses

In the additional exploratory analyses that were conducted (but not pre-registered), three channels were identified as showing significant block type effects but no significant main effect of time in either the HbO_2 or the HHb signal. These are Channel 8 (right inferior parietal), Channel 20 (right IFG), and Channel 31 (right DLPFC). The HRF plots for these additional three channels can be found in **Supplementary Figure *4***, **Supplementary Figure *5***, and **Supplementary Figure *6*** below. Exploratory paired t-tests were conducted on these three channels to investigate the time course of these block type effects; results are reported in **Supplementary Supplementary Table *16***. No significant associations were found when conducting exploratory individual differences correlation analyses on data from these three channels: Channel 8, bins 4, 5, and 7; p = .130, Channel 20, bin 1; p = .920, Channel 33, all bins; p = .770.

Supplementary Table 16. Time course of the exploratory block type effects.

Location	Channel	Signal	0 – 5s	5 – 10s	10 – 15s	15 – 20s	20 – 25s	25 – 30s	30 – 35s
**Right inferior parietal**	8	HHb	*t* (35) = 1.232, *p* = .226, *d* = .205	*t* (35) = 1.879, *p* = .069, *d* = .313	*t* (35) = 1.594, *p* = .120, *d* = .266	***t* (35) = 2.328, *p* = .026, *d* = .046**	***t* (35) = 2.030, *p* = .050, *d* = .338**	*t* (35) = 1.495, *p* = .144, *d* = .249	***t* (35) = 2.410, *p* = .021, *d* = .402**
**Right IFG**	20	HHb	***t* (41) = 3.199, *p* = .003**, *d* = .494**	*t* (41) = 2.000, *p* = .052, *d* = .309	*t* (41) = 1.079, *p* = .287, *d* = .166	*t* (41) = 1.632, *p* = .110, *d* = .252	*t* (41) = 1.139, *p* = .261, *d* = 176	*t* (41) = 1.309, *p* = .198, *d* = .202	*t* (41) = 1.702, *p* = .096, *d* = .263
**Right DLPFC**	31	HHb	***t* (42) = 3.143, *p* = .003**, *d* = .479**	***t* (42) = 2.881, *p* = .006**, *d* = .439**	***t* (42) = 2.450, *p* = .019**, *d* = .374**	***t* (42) = 3.512, *p* = .001**, *d* = .546**	***t* (42) = 2.957, *p* = .005**, *d* = .451**	***t* (42) = 2.541, *p* = .015**, *d* = 387**	***t* (42) = 3.177, *p* = .003**, *d* = .485**

Note. Statistically significant results are highlighted in bold. * = significant after correcting the FDR (7 comparisons). IFG = inferior frontal gyrus, DLPFC = dorsolateral prefrontal cortex.

Supplementary Figure 4. Haemodynamic response function for Channel 8 (right parietal).

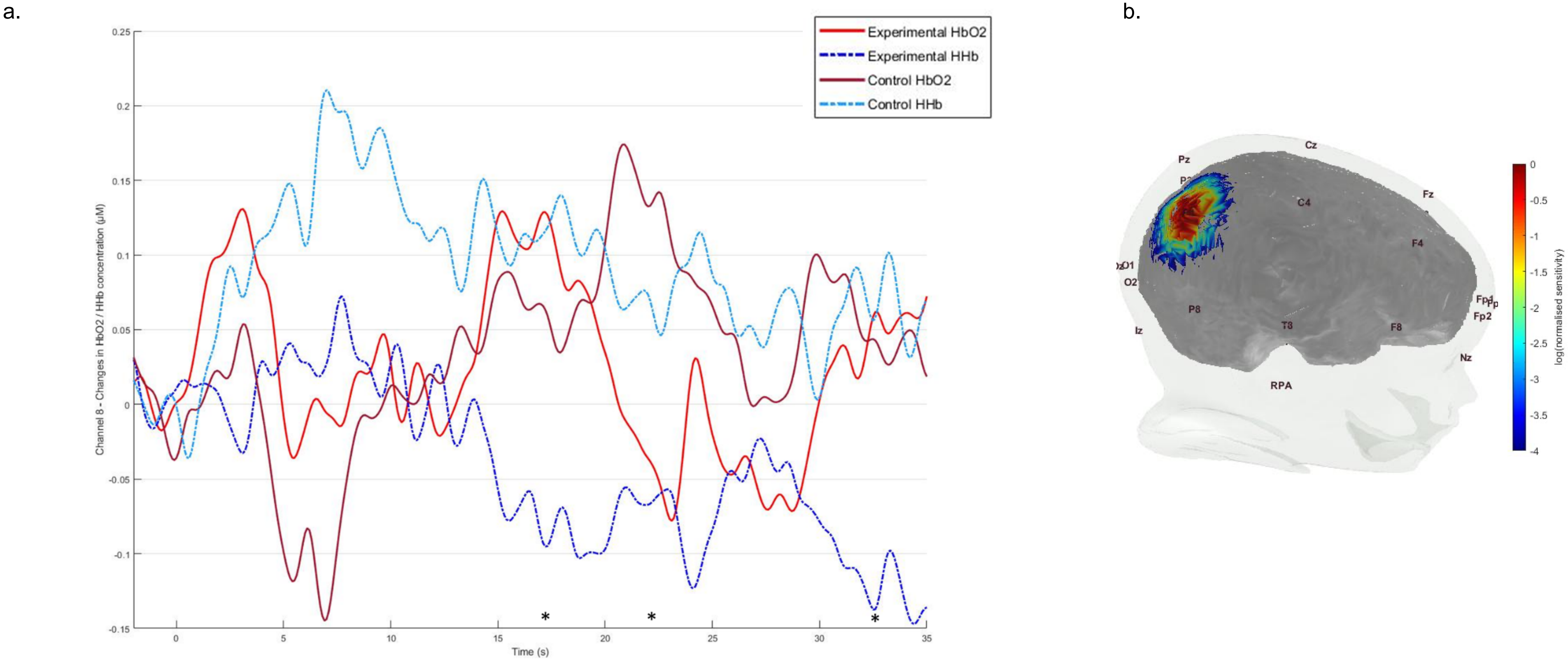

Note. a. Average haemodynamic response function for Channel 8 (*N* = 36) from -2 seconds (baseline) to 35 seconds of the block time-course. Asterisks denote time-bins where there is a significant block type effect (the significant effects in both time bins survived the procedure for controlling the FDR).
b. Sensitivity profile illustrating the position of Channel 8 on the cortex and the channel sensitivity (heat map: red = more sensitive, blue = less sensitive).

Supplementary Figure 5. Haemodynamic response function for Channel 20 (right inferior frontal gyrus, triangular).

a.

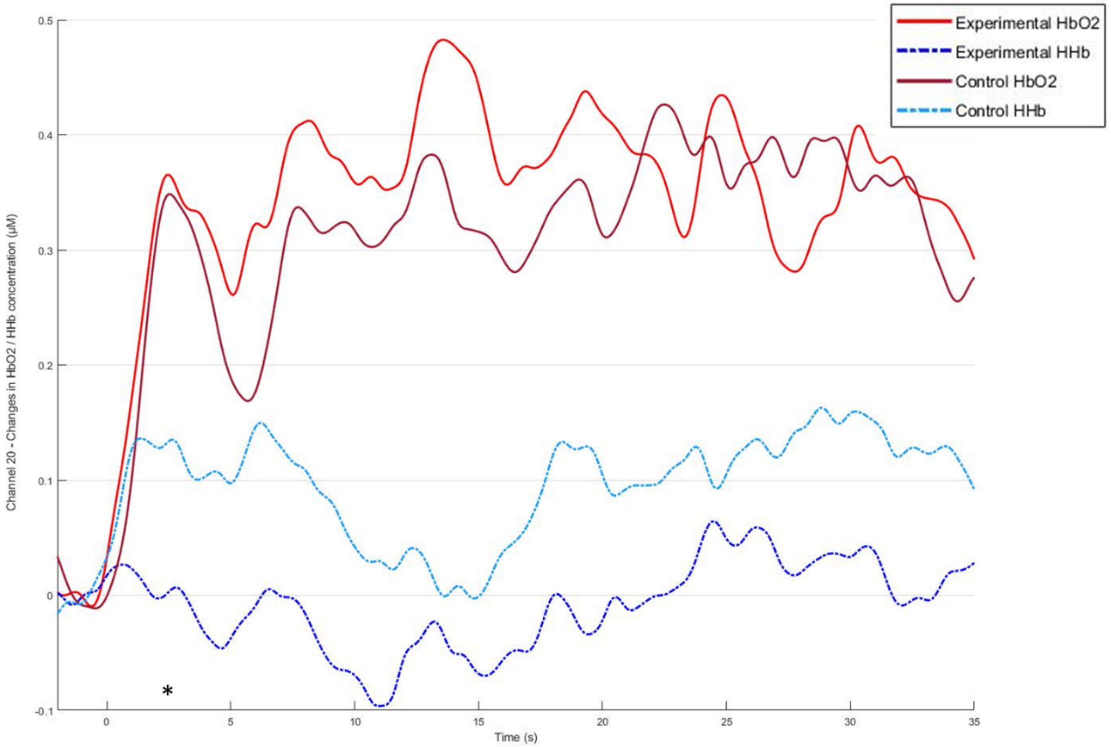

b.

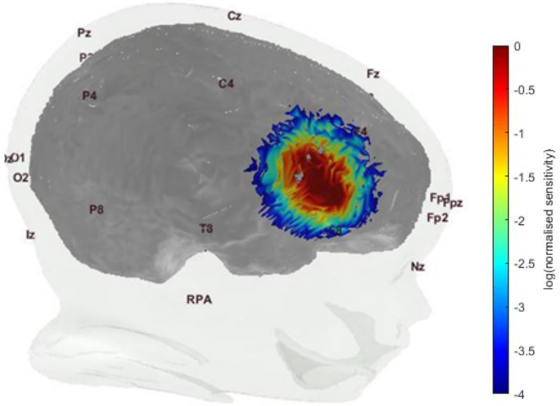

Note. a. Average haemodynamic response function for Channel 20 (*N* = 42) from -2 seconds (baseline) to 35 seconds of the block time-course. Asterisks denote time-bins where there is a significant block type effect (the significant effect in this time bin survived the procedure for controlling the FDR).
b. Sensitivity profile illustrating the position of Channel 20 on the cortex and the channel sensitivity (heat map: red = more sensitive, blue = less sensitive).

Supplementary Figure 6. Haemodynamic response function for Channel 31 (right inferior frontal gyrus, orbital).

a. b.

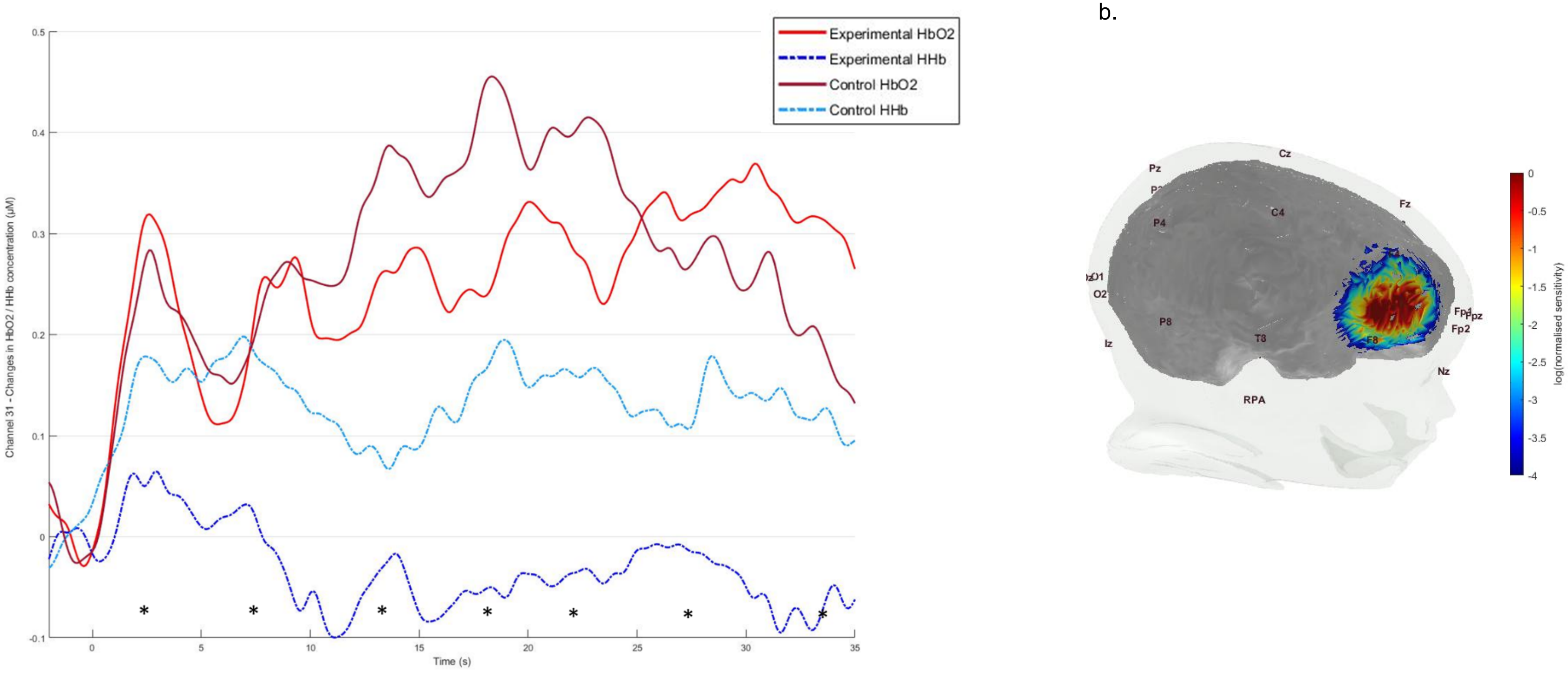

Note. a. Average haemodynamic response function for Channel 31 (N = 43) from -2 seconds (baseline) to 35 seconds of the block time-course. Asterisks denote time-bins where there is a significant block type effect (the significant effects in all time bins survived the procedure for controlling the FDR).
b. Sensitivity profile illustrating the position of Channel 31 on the cortex and the channel sensitivity (heat map: red = more sensitive, blue = less sensitive).

8. Exploratory Longitudinal Brain Associations from 10- to 16-months

As discussed in the manuscript, it was stated in the pre-registered analysis plan that preliminary exploratory analyses would be conducted on the longitudinal fNIRS data to examine change in brain activation across assessment points. However, due to limited statistical power of these analyses (only 18 participants had valid fNIRS data at both assessment points), we have only provided a summary of results in the manuscript. The full results are reported here, but it should be noted that results should be interpreted with caution as analyses are both exploratory and limited in statistical power.

8.1. Channels Showing Significant Block Type Effects at 16-months

Overall, nine channels (including the three channels found in the non-preregistered exploratory analyses, reported in Section 3.4. above) showed significant block type effects at 16-months. Separate linear mixed models (restricted maximum likelihood estimation) were conducted on these nine channels with assessment point (10-months, 16-months) as a fixed factor and participants as a random factor. For channels showing significant effects in the HbO_2 signal, the variable was the HbO_2 difference score (calculated as HbO_2 in experimental blocks minus HbO_2 in control blocks) across the block time course. For channels showing significant effects in the HHb signal, the variable was the HHb difference score (calculated as HHb in control blocks minus HHb in experimental blocks) across the block time course. The models were tested to investigate the change in activation from the 10-month assessment point to the 16-month assessment point (note that Channel 26 (HbO_2) and Channel 33 (HHb) showed significant block type effects at both assessment points).

The results are reported in

Supplementary Table *17* below, and estimated marginal means are reported in **Supplementary Table 18**. Results indicated that activation (HHb difference, i.e., greater HHb *decrease* in experimental compared to control blocks) in Channel 8 (right inferior parietal), Channel 28 (left DLPFC) and Channel 31 (right IFG, orbital) increased significantly from 10- to 16-months; see **Supplementary Figure 7**. The results of this exploratory analysis did not survive the correction for the FDR (N = 9 comparisons) and so can only be considered preliminary.

Supplementary Table 17. Exploratory longitudinal brain changes in channels with a significant block type effect at 16-months.

Location	Channel	Signal	Test Statistic	*p*	Effect size (Cohen's *d*)
L superior parietal	6	HHb	*F* (1, 87.116) = 1.286	.260	.225
R inferior parietal	8	HHb	*F* (1, 88.693) = 4.068	**.047**	.399
R IFG	20	HHb	*F* (1, 92.062) = 1.670	.199	.256
R IFG	23	HHb	*F* (1, 86.591) = 2.078	.153	.286
L DLPFC	28	HHb	*F* (1, 92.284) = 4.096	**.046**	.401
R DLPFC	26	HbO_2	*F* (1, 79.220) = .007	.934	.017
R IFG, orbital	31	HHb	*F* (1, 97.660) = 5.127	**.026**	.448
L OFC	29	HHb	*F* (1, 97.256) = 1.213	.274	.218
R OFC	33	HHb	*F* (1, 91.008) = .358	.551	.119

Note. Statistically significant results are highlighted in bold. For all channels (except Channel 26), the variable was the HHb difference score (calculated as HHb in control blocks minus HHb in experimental blocks) across the block time course. For Channel 26, the variable was the HbO_2 difference score (calculated as HbO_2 in experimental blocks minus HbO_2 in control blocks) across the block time course.
L = left, R = right. IFG = inferior frontal gyrus, DLPFC = dorsolateral prefrontal cortex, OFC = orbital frontal cortex.

Supplementary Table 18. Exploratory longitudinal brain associations: estimated marginal means (channels that showed a significant block type effect at 16-months, but not at 10-months).

Location	Channel	Assessment Point	Estimated marginal mean	Standard error	df	Confidence interval
L superior parietal gyrus (HHb)	6	10-months	.333	.495	51	-.661, 1.327
		16-months	1.127	.495	38	.125, 2.129
R inferior parietal (HHb)	8	10-months	-.452	.608	56	-1.670, .765
		16-months	1.220	.564	35	.075, -2.366
R IFG (HHb)	20	10-months	.199	.446	56	-.694, 1.092
		16-months	1.046	.480	41	.076, 2.016

R IFG (HHb)	23	10-months	.097	.376	58	-.655, .849
		16-months	.969	.474	42	.012, 1.926
L DLPFC (HHb)	28	10-months	.043	.410	57	-.778, .863
		16-months	1.267	.445	41	.368, 2.165
R DLPFC (HbO_2)	26	10-months	1.011	.949	56	-.889, 2.912
		16-months	1.135	1.132	36	-1.161, 3.431
R IFG, orbital (HHb)	31	10-months	.216	.434	57	-.654, 1.085
		16-months	1.581	.419	42	.736, 2.426
L OFC (HHb)	29	10-months	.142	.399	57	-.657, .941
		16-months	.741	.369	41	-.005, 1.487
R OFC (HHb)	33	10-months	.684	.458	58	-.233, 1.601
		16-months	1.105	.523	42	.029, 2.180

Note. L = left, R = Right. IFG = inferior frontal gyrus, DLPFC = dorsolateral prefrontal cortex, OFC = orbital frontal cortex.

Supplementary Figure 7. Exploratory longitudinal brain changes (channels showing significant increase in activation from 10- to 16-months).

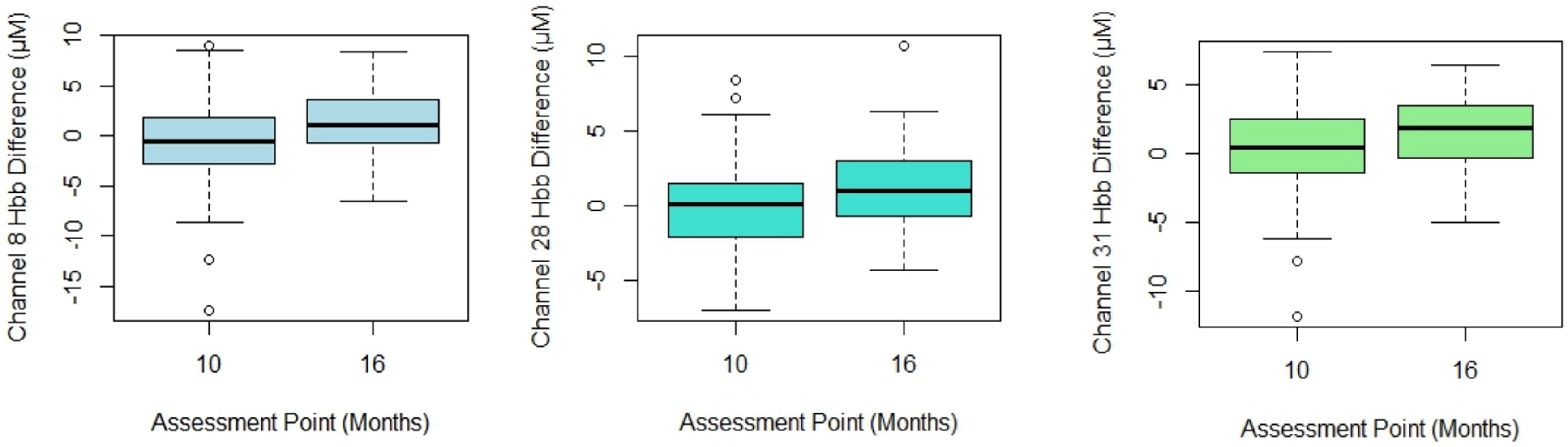

Note. This figure illustrates the increase in activation from the 10-month assessment point to the 16-month assessment point in channels showing a significant effect of assessment point (i.e., significant change over time): Channel 8 (right inferior parietal cortex), Channel 28 (left dorsolateral prefrontal cortex), Channel 31 (right inferior frontal gyrus, orbital). Note that all three channels showed a significant block type effect at 16-months, but not at 10-months. The variables on the y-axis refer to the HHb difference score (calculated as HHb in control blocks minus HHb in experimental blocks) across the block time course.
10 = 10-months, 16 = 16-months. HHb = Deoxygenated haemoglobin.

8.2. Channels Showing Significant Block Type Effects at 10-months

Six additional linear mixed models were conducted to investigate the change in activation across assessment points in channels showing significant effects at 10-months, but not at 16-months. Results (**Supplementary Table 19**, estimated marginal means are reported in **Supplementary Table 20**) indicated that activation (HbO_2 difference) in Channel 25 decreased significantly from 10- to 16-months; see **Supplementary Figure 8**. The result of this exploratory analysis is preliminary and did not survive the correction for the FDR (*N* = 6 comparisons), and so should be considered cautiously.

Supplementary Table 19. Exploratory longitudinal brain changes in channels with a significant block type effect at 10-months.

Location	Channel	Signal	Test Statistic	*p*	Effect size (Cohen's *d*)
R inferior parietal	10	HbO_2	$F\,(1, 75.167) = .272$	.603	.103
R superior parietal	12	HbO_2	$F\,(1, 75.753) = .229$	.634	.095
R DLPFC	25	HbO_2	$F\,(1, 94.017) = 4.377$	**.039**	.414
	25	HHb	$F\,(1, 81.980) = .490$	.486	.139
R OFC	32	HbO_2	$F\,(1, 75.985) = .056$	.814	.047
	32	HHb	$F\,(1, 77.131) = .001$	.976	.006

Note. Statistically significant results are highlighted in bold. L = left, R = Right. DLPFC = dorsolateral prefrontal cortex, OFC = orbital frontal cortex.

Supplementary Table 20. Exploratory longitudinal brain associations: estimated marginal means (change in channels that showed a significant block type effect at 10-months).

Location	Channel	Assessment Point	Estimated marginal mean	Standard error	df	Confidence interval
R inferior parietal (HbO_2)	10	10-months	2.215	.877	51	.454, 3.976
		16-months	1.590	.814	28	-.076, 3.257
R superior parietal (HbO_2)	12	10-months	1.955	.865	50	.218, 3.692
		16-months	1.416	.724	27	-.069, 2.901
R DLPFC (HbO_2)	25	10-months	1.652	.812	55	.025, 3.278
		16-months	-.748	.870	41	-2.384, .888
R DLPFC (HHb)	25	10-months	1.223	.398	55	.426, 2.020
		16-months	.764	.521	41	-.287, 1.816
R OFC (HbO_2)	32	10-months	.160	.721	56	-1.283, 1.604
		16-months	.468	10.88	42	-1.727, 2.663
R OFC (HHb)	32	10-months	1.222	.432	56	.359, 2.85
		16-months	1.245	.637	42	-.040, 2.530

Note. L = left, R = Right. IFG = inferior frontal gyrus, DLPFC = dorsolateral prefrontal cortex, OFC = orbital frontal cortex.

Supplementary Figure 8. Exploratory longitudinal brain change (significant block type effects at 10-months but not at 16-months).

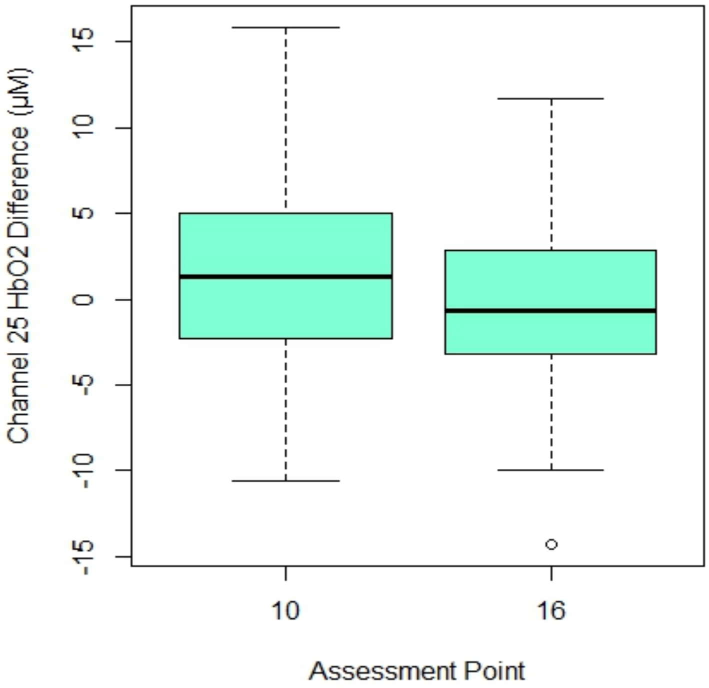

Note. This figure illustrates the decrease in activation from the 10-month to the 16-month assessment point in Channel 25 (HbO_2), which showed significant change over time. The y-axis refers to the HbO_2 difference score (calculated as HbO_2 in experimental blocks minus HbO_2 in control blocks) across the block time course. Channel 25 covers the right dorsolateral prefrontal cortex. 10 = 10-months, 16 = 16-months.

9. References

Aasted, C. M., Yücel, M. A., Cooper, R. J., Dubb, J., Tsuzuki, D., Becerra, L., Petkov, M. P., Borsook, D., Dan, I., & Boas, D. A. (2015). Anatomical guidance for functional near-infrared spectroscopy: AtlasViewer tutorial. *Neurophotonics*, *2*(2), 020801. https://doi.org/10.1117/1.nph.2.2.020801

Boas, D. A., & Dale, A. M. (2005). Simulation study of magnetic resonance imaging–guided cortically constrained diffuse optical tomography of human brain function. *Applied Optics*, *44*(10), 1957–1968. https://doi.org/10.1364/AO.44.001957

Boas, D. A., Dale, A. M., & Franceschini, M. A. (2004). Diffuse optical imaging of brain activation: Approaches to optimizing image sensitivity, resolution, and accuracy. *NeuroImage*, *23*(SUPPL. 1), S275–S288. https://doi.org/10.1016/j.neuroimage.2004.07.011

Brigadoi, S., Galderisi, A., Pieropan, E., Cooper, R. J., Cutini, S., Baraldi, E., Cobelli, C., Dell'Acqua, R., Sparacino, G., & Trevisanuto, D. (2019). Mapping hemodynamic changes during hypoglycemia in the very preterm neonatal brain: Preliminary results. *Optics InfoBase Conference Papers*, *Part F142-ECBO 2019*, 11074_13. https://doi.org/10.1117/12.2526974

Collins-Jones, L. H., Arichi, T., Poppe, T., Billing, A., Xiao, J., Fabrizi, L., Brigadoi, S., Hebden, J. C., Elwell, C. E., & Cooper, R. J. (2021). Construction and validation of a database of head models for functional imaging of the neonatal brain. *Human Brain Mapping*, *42*(3), 567–586. https://doi.org/10.1002/hbm.25242

Fang, Q., & Boas, D. A. (2009). Tetrahedral mesh generation from volumetric binary and grayscale images. *Proceedings - 2009 IEEE International Symposium on Biomedical Imaging: From Nano to Macro, ISBI 2009*, 1142–1145. https://doi.org/10.1109/ISBI.2009.5193259

Fiske, A., de Klerk, C., Lui, K. Y. K., Collins-Jones, L., Hendry, A., Greenhalgh, I., Hall, A., Scerif, G., Dvergsdal, H., & Holmboe, K. (2022). The neural correlates of inhibitory control in 10-month-old infants: A functional near-infrared spectroscopy study. *NeuroImage*, *257*. https://doi.org/10.1016/J.NEUROIMAGE.2022.119241

Frijia, E. M., Billing, A., Lloyd-Fox, S., Vidal Rosas, E., Collins-Jones, L., Crespo-Llado, M. M., Amadó, M. P., Austin, T., Edwards, A., Dunne, L., Smith, G., Nixon-Hill, R., Powell, S., Everdell, N. L., & Cooper, R. J. (2021). Functional imaging of the developing brain with wearable high-density diffuse optical tomography: A new benchmark for infant neuroimaging outside the scanner environment. *NeuroImage*, *225*(October). https://doi.org/10.1016/j.neuroimage.2020.117490

Hendry, A., Greenhalgh, I., Bailey, R., Fiske, A., Dvergsdal, H., & Holmboe, K. (2021). *Development of directed global inhibition, competitive inhibition and behavioural inhibition during the transition between infancy and toddlerhood*. https://doi.org/10.31234/OSF.IO/MHKAJ

Holmboe, K., Larkman, C., Klerk, C. de, Simpson, A., Bell, M. A., Patton, L., Christodoulou, C., & Dvergsdal, H. (2021). The Early Childhood Inhibitory Touchscreen Task: A new measure of response inhibition in toddlerhood and across the lifespan. *PloS One*, *16*(12). https://doi.org/https://doi.org/10.1371/journal.pone.0260695

Huppert, T. J., Diamond, S. G., Franceschini, M. A., & Boas, D. A. (2009). HomER: A review of time-series analysis methods for near-infrared spectroscopy of the brain. *Applied Optics*, *48*(10). https://doi.org/10.1364/AO.48.00D280

Jenkinson, M., Pechaud, M., & Smith, S. (2005). BET2-MR-Based Estimation of Brain, Skull and Scalp Surfaces. *Human Brain Mapping*, *17*(2), 143–155. www.fmrib.ox.ac.uk/analysis/research/bet

Lloyd-Fox, S., Blasi, A., & Elwell, C. E. (2010). Illuminating the developing brain: The past, present and future of functional near infrared spectroscopy. *Neuroscience and Biobehavioral Reviews*, *34*(3), 269–284. https://doi.org/10.1016/j.neubiorev.2009.07.008

Lui, K. Y. K., Hendry, A., Fiske, A., Dvergsdal, H., & Holmboe, K. (2021). Associations between touchscreen exposure and hot and cool inhibitory control in 10-month-old infants. *Infant Behavior and Development*, *65*, 101649. https://doi.org/10.1016/J.INFBEH.2021.101649

Schweiger, M., & Arridge, S. (2014). The Toast++ software suite for forward and inverse modeling in optical tomography. *Journal of Biomedical Optics*, *19*(4), 040801. https://doi.org/10.1117/1.jbo.19.4.040801

Shi, F., Yap, P.-T., Wu, G., Jia, H., Gilmore, J. H., Lin, W., & Shen, D. (2011). Infant Brain Atlases from Neonates to 1- and 2-Year-Olds. *PLoS ONE*, *6*(4), e18746. https://doi.org/10.1371/journal.pone.0018746

Taga, G., Homae, F., & Watanabe, H. (2007). Effects of source-detector distance of near infrared spectroscopy on the measurement of the cortical hemodynamic response in infants. *NeuroImage*, *38*(3), 452–460. https://doi.org/10.1016/J.NEUROIMAGE.2007.07.050
